# Suicide and psychiatric disorders associated with amphetamine type stimulant use: a systematic review and meta-analysis

**DOI:** 10.3389/fpsyt.2026.1654091

**Published:** 2026-03-13

**Authors:** Halima Mohammed Adam, Ahmed Hamed Aljadani, Mohamed Abouzed, Benayan Bani Alrasheedy, Muath Abdulaziz Alarfaj

**Affiliations:** 1 Psychology Department, Education College, University of Ha’il, Hail, Saudi Arabia; 2 College of Medicine, University of Ha’il, Hail, Saudi Arabia; 3 Department of Psychiatry, College of Medicine, Al-Azhar University, Cairo, Egypt; 4 Psychological Rehabilitation Department, Eradah Complex for Mental Health, Hail, Saudi Arabia

**Keywords:** amphetamine, methamphetamine, stimulant, substance use disorder, suicide

## Abstract

**Objective:**

This study reviews the literature to determine the prevalence and nature of suicidality and psychiatric comorbidity in individuals with amphetamine-type stimulant (ATS) use to inform targeted interventions.

**Methods:**

We searched the Web of Science, PubMed, Scopus, and Cochrane Library databases for relevant papers published until March 2025. Two independent reviewers collected the following data: baseline information, outcomes, prevalence of suicidality, psychiatric disorders, employment status, marital status, additional substances used, age at onset of use, and duration of use.

**Results:**

We collected 2,969 records after excluding 2,072 duplicates. Thorough screening yielded 70 entries eligible for inclusion. Our analysis revealed prevalence rates of 26% for depression, 22% for hallucinations, 20% for suicidality, 23% for suicidal ideation, 17% for suicide attempts, and 13% for deaths by suicide. Additionally, 38% of individuals who use ATS also used multiple other substances concurrently, and the mean duration of ATS use was estimated to be 5.13 years. The total pooled sample size across all included studies was 311,669 persons.

**Conclusion:**

This systematic review highlights the high prevalence of psychiatric conditions, including depression, hallucinations, and suicidality, frequently co-occurring with concurrent use of multiple substances and psychiatric disorders. These findings represent prevalence rates within the ATS-using population, and no comparison with non-ATS populations was performed; therefore, they do not imply an excess or attributable risk. The results underscore the critical need for integrated screening and service-planning guidance in this vulnerable population.

## 1 Introduction

The number of people attempting suicide has been steadily rising worldwide. Suicide is responsible for 1.4% of premature deaths, and it is the second most common cause of death among young adults in their 20s and 30s (1). Globally, approximately 700,000 suicide deaths have been reported. According to these statistics, at least two people attempt to take their own lives every day. Regrettably, many of these attempts result in death (2). Although suicide is not classified as a standalone diagnosis, it was included in the DSM-5 under the chapter on conditions of clinical concern. This inclusion aims to highlight risk factors and improve clinical practices for the prevention of suicide. Suicide is considered a comorbid condition with several mental disorders, such as depression, schizophrenia, personality disorders, and substance use disorders (3–5). Suicidal ideation, plans, attempts, and deaths by suicide are all included in the broad category of suicidality. The desire to die is the primary difference between suicide and self-harming behaviors (6).

A major concern is the association between amphetamine-type stimulants (ATS) and suicide. Deaths involving amphetamines, with or without opioids, have been rising across all population segments (7). Complex interactions among biological, social, and psychological factors have contributed to a sharp rise in ATS use worldwide (8). This trend has become a significant public health concern. Amphetamine-type stimulants (ATS) are powerful central nervous system stimulants. They have drawn attention not only for their addictive properties but also for their link to numerous negative health outcomes, including a high rate of deaths from stimulant overdose (9). Amphetamines and suicidality, fueled by substantial public health consequences of substance use patterns, have been under more scrutiny. Several factors may explain the association between amphetamine use and increased risk of suicidal thoughts and behaviors. These include specific pharmacological effects, physical injuries, comorbid psychiatric disorders, and sociocultural influences (10). In epidemiological studies on amphetamine-related suicide deaths, demographic factors have been observed to play a substantial role.

One study among adolescents revealed that many adolescents who used drugs reported suicidal thoughts or attempts. The study also found statistically significant associations between suicide attempts and amphetamine use (11). Gender plays a key role in the demographic factors affecting suicidality among individuals who use amphetamines. Men are more likely than women to exhibit suicidal tendencies. This difference may be linked to social expectations regarding emotional expression and seeking support (12).

Over the past few decades, methamphetamine use has increased considerably and, according to evidence, it has become more common among various population groups, particularly young and socially disadvantaged populations. A range of social factors contribute to this escalation, including poor parent-child relationships (13, 14). The risk is further amplified by socioeconomic challenges such as poverty, unemployment, and social isolation. Additional determinants include involvement in criminal activities, chronic health conditions such as hepatitis B and C, HIV infection, and psychiatric disorders, particularly depression (7, 13). Several factors have been associated with high amphetamine-related suicide risk, including depression, aggressive behavior, and social dysfunction. All these factors are exacerbated by early exposure to substances such as amphetamines (10). Risk factors for suicide attempts and deaths by suicide, including impulsiveness and aggression, have also been reported (15–18).

Further, individuals who use methamphetamine along with other substances are more likely to die by suicide than those using methamphetamine alone (19). Additionally, comorbidities, particularly schizophrenia and mood disorders, significantly increase the risk of suicide attempts among individuals who use amphetamines. For instance, individuals with schizophrenia who also use substances such as amphetamines face multiple challenges. These include pronounced mood dysregulation and poor medication adherence, both of which significantly increase the risk of suicide (20).

The neurological and biological effects of amphetamines further complicate the relationship between suicide and substance use. These substances alter reward pathways and disrupt neurotransmitter systems. The drug causes the brain to release a higher amount of dopamine, which disrupts the sympathetic nervous system (SNS) and derails mitochondrial and endoplasmic reticulum (ER) processes (21). Methamphetamine intoxication occurs due to several mechanisms. These include disruption of the blood-brain barrier, increased neurotransmitter discharge, blockage of uptake transporters, and receptor degeneration. The resultant oxidative stress gives rise to both intense euphoria and severe neurotoxicity, expressed primarily as neuroinflammation and oxidative stress. Severe manifestations of cardiovascular and cerebrovascular diseases, such as Parkinson’s and Alzheimer’s diseases, are caused by these acute stimuli (21, 22).

Vrajová et al. (2021) demonstrated that individuals who use ATS experience a range of emotional mental health problems and disruptions in sleep–wake patterns. These include depression, anxiety, hyperactivity, aggression, psychosis, and sleep disorders (23). People who use ATS undergo chemical changes in the brain. These changes lead to emotional instability, which increases suicide risk among individuals with mental health disorders (24). People who experience changes in their mood control gene expressions present an increased risk of suicide (25, 26).

Exposure to ATS causes long-term increases in reward-seeking behavior. This heightened reactivity to rewards and related cues contributes to a greater probability of drug relapse (27). Long-term use has been linked to deficits in language, motor abilities, information processing, memory, and cognition (28).

Social and environmental factors play a role in the development of suicidality among individuals who use ATS. Evidence suggests that early traumatic events combined with inherited mental disorders in family members significantly increase the risk of substance use. They also heighten the likelihood of suicidal thoughts and behaviors. The relationship between childhood adversities and family support suggests that targeted interventions in these areas could help prevent suicide among individuals who use amphetamines (29, 30).

Our systematic analysis evaluates existing medical research on ATS use and its relationship with suicidality. It also identifies key research themes, information gaps, and potential opportunities for future studies. Our research aims to develop a more advanced understanding of the connection between ATS use and suicidality by analyzing multiple studies. This review explores multiple aspects of the relationship between ATS use and suicidal thoughts and actions. These include associations with other mental health problems and links to environmental and social conditions. The focus of our review is to determine the prevalence of suicidality and suicidal behaviors, including ideation and attempts, as well as deaths attributed to ATS use disorder. Additionally, we examine related factors such as concurrent use of multiple substances, psychiatric disorders (including depression and hallucinations), and the duration of ATS use.

## 2 Methods

This review followed the formatting guidelines of the Cochrane Handbook for Systematic Reviews of Interventions and the Preferred Reporting Items for Systematic Reviews and Meta-Analyses (PRISMA) (31, 32).

### 2.1 Eligibility criteria

For the purpose of this meta-analysis, Amphetamine-Type Stimulants (ATS) primarily included amphetamine, methamphetamine, and their derivatives. We acknowledge the heterogeneity in the literature. Studies were included if the primary substance was ATS. Studies where the primary substance was Ecstasy/MDMA or other non-amphetamine stimulants (e.g., cathinones) were excluded unless the sample was clearly defined as individuals using multiple substances where ATS was a significant component (Tier 3 studies). We have carefully reviewed the included studies to ensure that the majority of the pooled sample represents individuals with a confirmed ATS use disorder or significant ATS exposure. This approach addresses the concern that our findings might reflect ‘stimulant use broadly’ by focusing the inclusion criteria on the core ATS group, while transparently acknowledging the inclusion of mixed-stimulant samples in the broader context of concurrent use of multiple substances.

We included studies in which suicidality was explicitly described as temporally or clinically associated with ATS use, such as during active use, withdrawal, or hospitalization for ATS-induced psychiatric symptoms. When this association was not directly stated, suicidality data were extracted only from participants with a confirmed diagnosis of ATS use disorder or documented ATS use within the study’s inclusion criteria. We acknowledge that this approach may capture suicidality that is not causally attributable to ATS use, but rather reflects comorbid psychiatric conditions or other confounders within ATS-using populations.

The inclusion criteria were as follows:

- Research focused on individuals with amphetamine or related substance use disorder
- Data obtained from hospital records or surveys
- Articles published in English
- Full-text articles were available for retrieval

The exclusion criteria were as follows:

- Studies where the prevalence of amphetamine-type stimulant (ATS)-related suicide could not be determined
- Reviews, letters to editors, comments, opinions, randomized controlled trials (RCTs), and abstracts
- Articles in non-English languages

### 2.2 Search strategy and information sources

The procedure for retrieving records was divided into several stages, as follows:

Preliminary publications were found through a general search conducted using generic phrases across PubMed, Scopus, and Embase. Subsequently, a more sophisticated approach utilizing MeSH terms and associated keywords was methodically implemented for the Web of Science, PubMed, Scopus, and Cochrane Library databases. Following are the search terms used: (Methamphetamine OR Deoxyephedrine OR Desoxyephedrine OR “N Methylamphetamine” OR Metamfetamine OR Desoxyn OR Metamfetamin* OR Stimulex OR Benzeneethanamine* OR Phenethylamine* OR dextromethamphetamine OR Meth OR Shabu) AND (self-murder OR suicid* OR Self-destruction OR Death OR Suicide). Our final search was conducted in March 2025 using a specific and detailed query. Additionally, references and citations in the collected records were carefully checked and manually reviewed to identify other relevant studies.

### 2.3 Selection method

The studies were screened by two independent, blinded reviewers. Screening involved evaluating research titles and abstracts based on specific criteria. To minimize bias, identifiable authorship information was concealed. A third reviewer resolved any discrepancies between the initial reviewers. After completing the initial screening phase, the two reviewers conducted a full-text review of the papers that met the criteria to verify their eligibility and compared the results. To avoid duplication of data, we carefully cross-referenced author names, study locations, and recruitment periods. Where potential overlapping samples were identified, we prioritized the study with the most comprehensive data on suicidality and excluded the others.

### 2.4 Data collection

Using a predefined set of variables, two independent reviewers employed a two-step method to extract data from full-text articles into a shared Google spreadsheet. They collected baseline data in the first step, such as the author’s last name, study design, the author’s country of origin, and results. Additionally, they gathered information on the number of patients, their age, distribution of genders, employment status, marital status, additional substances used, and age at the onset of ATS use. For analysis, they extracted data on the prevalence of ATS-related factors, depression, hallucinations, suicidal ideation, suicide attempts, deaths by suicide, duration of ATS use, and prevalence of concurrent use of multiple substances. See **Supplementary Table 1**.

### 2.5 Outcome measures

Prevalence of psychiatric disorders: We pooled the prevalence of depression and hallucinations among individuals who use ATS at the time of presentation or interviewing in each study, as well as the types of hallucinations whenever clearly stated.

Amphetamine type Stimulant (ATS)-related suicidality: We gathered all the reported prevalence of suicidality among individuals who use ATS (suicidality, suicidal ideation, and suicide attempts) at the time of evaluation.

ATS-related deaths by suicide: This outcome represents the proportion of individuals whose death was attributed to ATS use disorder among all deaths in people with ATS use.

Concurrent use of multiple substances: Whenever provided, we documented the prevalence of concurrent use of drugs, alcohol, or other substances along with ATS.

Duration of ATS use: We assimilated the reported duration of ATS use and pooled them in our analysis to estimate the mean duration.

### 2.6 Sensitivity analysis

Explicit description of three-tier categorization methodology

Tier 1 (Explicit association, n=12): Studies explicitly documenting suicidality during active ATS use, intoxication, withdrawal, or hospitalization for ATS-induced psychiatric symptoms

Tier 2 (Inferred association, n=47): Studies reporting suicidality among participants with a confirmed diagnosis of ATS use disorder, but without explicit temporal documentation

Tier 3 (Weak association, n=10): Studies in polysubstance contexts where ATS was one of multiple substances used, limiting attribution specificity. See **Supplementary Table 2**.

### 2.7 Bias risk

All full-text publications were evaluated by two separate reviewers, and a third reviewer addressed any discrepancies. The National Institutes of Health Quality Assessment Tool for Observational and Cohort Studies was used to assess the methodological quality of each cohort study (33).

### 2.8 Statistical analysis

Data were analyzed using R version 4.3.3. Polysubstance use and psychiatric disorders were pooled, detailing the subtypes of hallucinations to avoid overlap. Additionally, the mean duration of ATS use was pooled. The combined means of absence duration and their 95% confidence intervals (CIs) were reported. Statistical significance was set at p < 0.05. The I² statistic was used to assess heterogeneity in the included trials. I² > 40% and a χ² p < 0.1 indicated significant heterogeneity among the included studies. To address the heterogeneity and variability of data, a random-effects model was applied. In addition, we considered meta-regression using the duration of ATS use whenever possible. The results included details of individual studies, and the diversity of effect measures was displayed using the forest plot. Heterogeneity (τ²) was estimated using the restricted maximum likelihood (REML) method.

## 3 Results

### 3.1 Study selection

**Figure 1:**
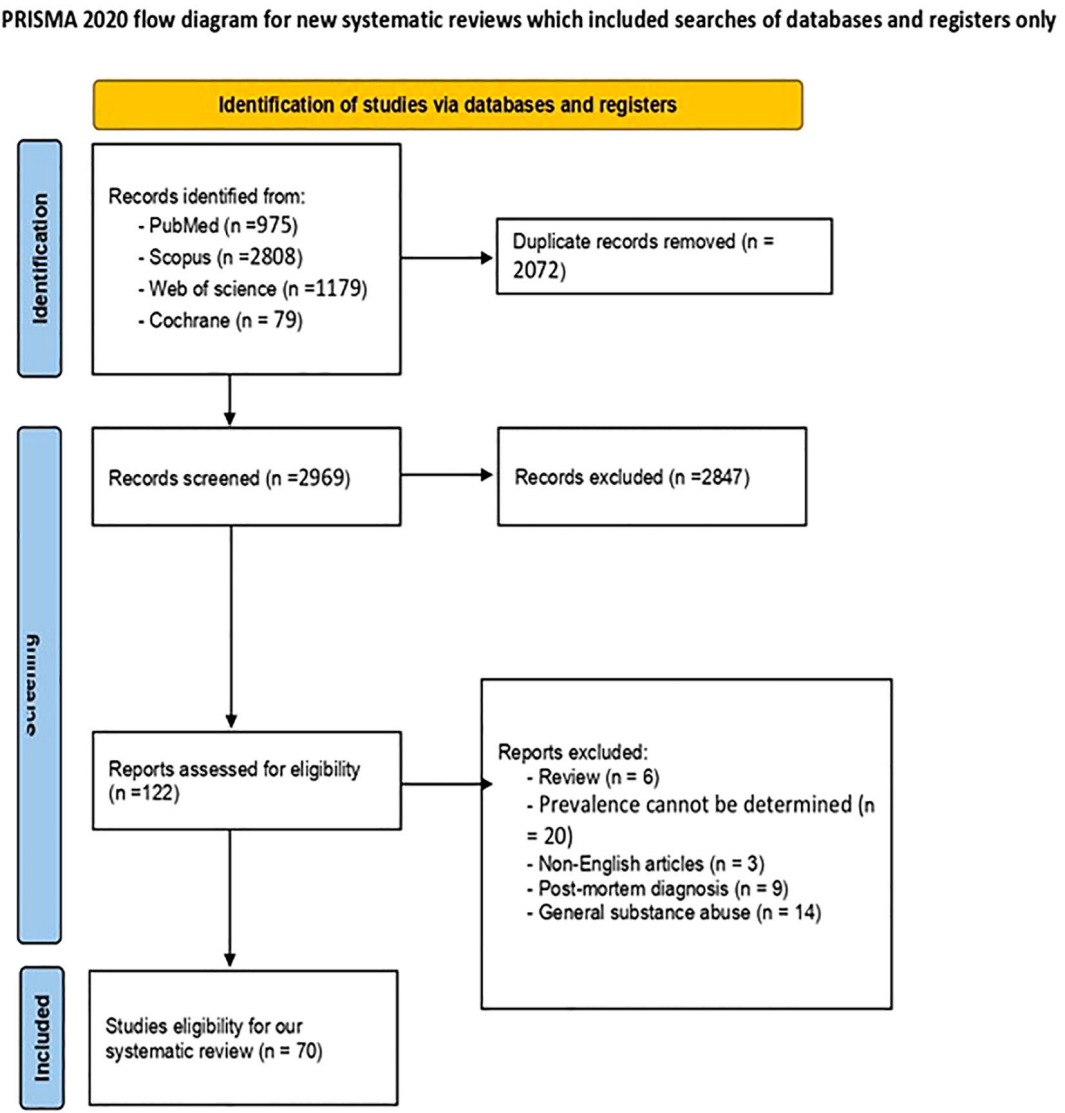
PRISMA flow diagram of the study selection process.

While searching the four databases, we found 5,041 records. After removing 2,072 duplicates, we obtained 2,969 unique records. We then checked the titles and abstracts, removing 2,847 entries. We extracted the complete texts of the remaining 122 records and evaluated them based on our eligibility criteria. During this examination, we excluded three non-English papers, six review articles, nine studies with postmortem diagnoses, 20 studies without prevalence estimates, and 14 studies that did not address amphetamine-type stimulant (ATS) as an addictive stimulant. Ultimately, 70 articles were included in this review. **Figure 1** presents the flow diagram for study selection.

### 3.2 Characteristics of included studies

The review included 70 studies (12, 19, 34, 35). Twenty-six were retrospective cohort studies, 10 were prospective cohort studies, 33 were cross-sectional studies, and one was a case-control study. Further, 26 reports were from the United States, six from Iran, another six from Taiwan, four from Canada, 12 from Australia, three from Japan, and one from the United Kingdom, among other countries. The total number of participants was 311,669, 45.27% of whom were female participants. The mean age of participants at the onset of substance use was 21.39 (± 6.19) years, whereas their age at first consultation averaged 31.59 (± 7.84) years. A complete depiction of baseline characteristics is presented in **Supplementary Table 3**.

### 3.3 Risk of bias

The reviewers used NIH criteria to rate cohort studies as “good,” “fair,” or “poor.” A “good” rating indicates that the study had the lowest chance of bias and delivered valid findings. A “fair” rating implies some bias, but the conclusions remain valid. A “poor” rating reflects severe prejudice. **Supplementary Table 4** shows that five of the examined studies were of good quality, 58 were of fair quality, and seven were of poor quality.

### 3.4 Results of syntheses

Results regarding the prevalence of psychiatric disorders are presented below.

#### 3.4.1 Depression

**Figure 2:**
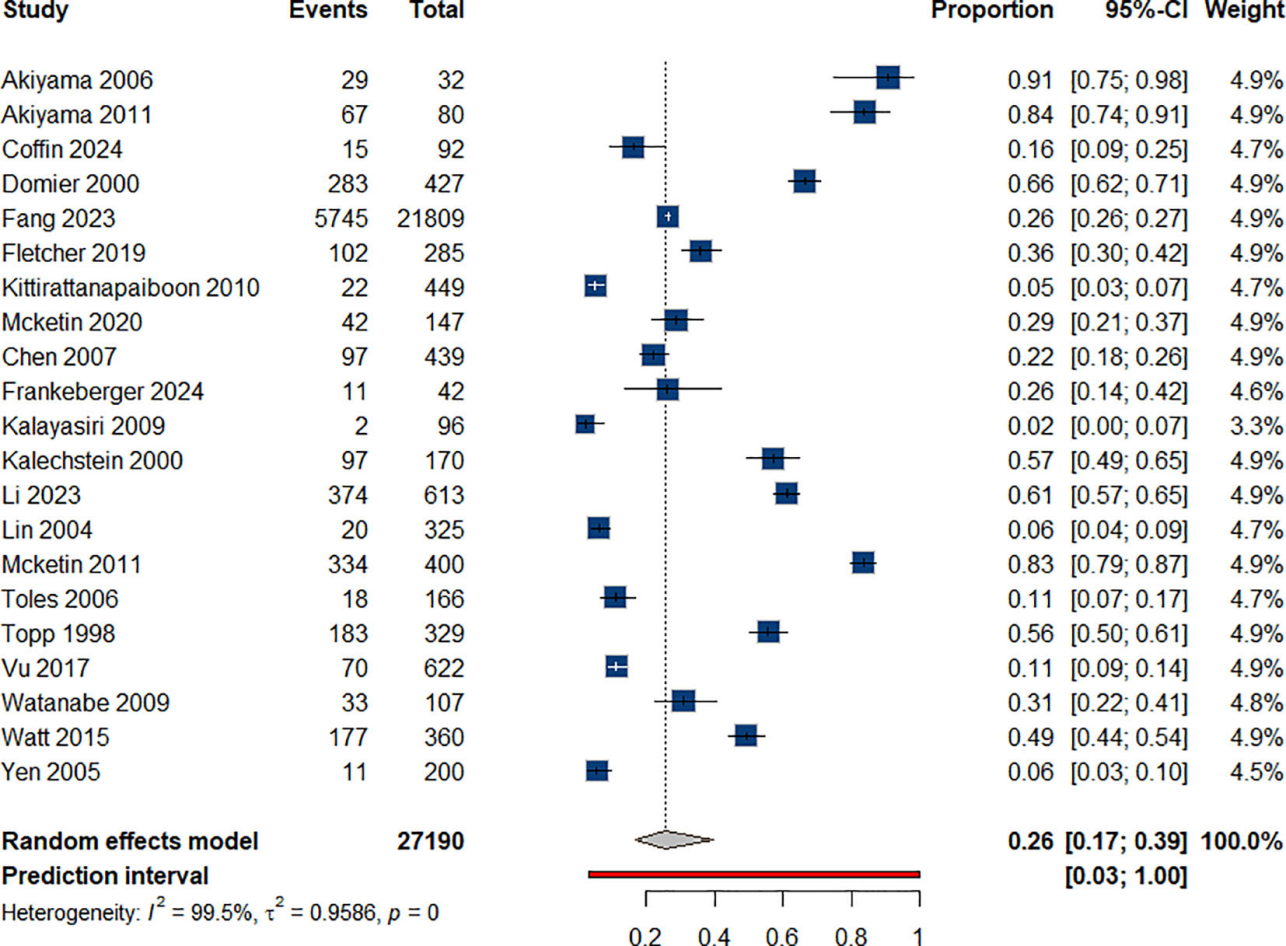
Prevalence of depression among individual with ATS use.

We estimated the prevalence of depression among patients who use amphetamines to be 26% (95% CI: 0.17-0.39), based on data presented in 17 studies:

(34, 36–51) **Figure 2**. Each year of ATS use significantly increased the odds of depression by 19% (OR = 1.19), **Table 1**.

**Table 1:** Meta-regression of the effect of addiction duration on suicide-related events.

Outcome	Predictor	Odds Ratio (OR)	95% CI for OR	Resolved I2	p-value
Suicidal Ideation	Intercept	0.09	0.05, 0.18	–	<.0001
(I²=92.17%)	Duration	1.21	1.09, 1.34	74.12%	0.0002
Suicide Attempts	Intercept	0.21	0.09, 0.52	–	0.0007
(I²=96.85%)	Duration	0.98	0.86, 1.11	0	0.732
Depression	Intercept	0.11	0.03, 0.36	–	0.0003
(I²=99.45%)	Duration	1.19	1.01, 1.41	28.32%	0.0427

#### 3.4.2 Hallucinations

**Figure 3:**
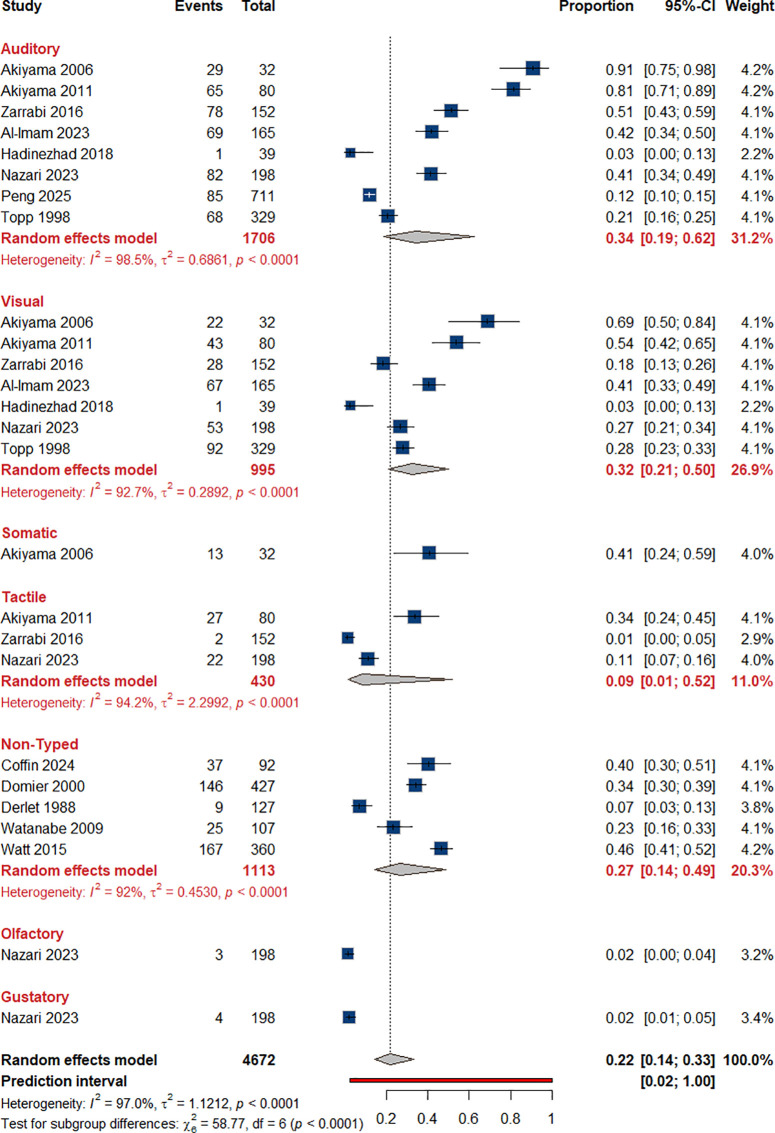
Prevalence of hallucinations among individual with ATS use.

Our analysis yielded a 22% (95% CI: 0.14-0.33) prevalence of hallucinations in general. We also examined the prevalence of several types of hallucinations. From eight studies (43, 51–57) we calculated the rate of auditory hallucinations to be 34% (95% CI: 0.19-0.62). Visual hallucinations were reported in sex studies (43, 51, 52, 55, 56, 58) Based on their data, we estimated the rate to be 32% (95% CI: 0.21-0.50). Additionally, the prevalence of tactile hallucinations was estimated to be 9% (95% CI: 0.01-0.52) (43, 53, 56). Meanwhile, five studies described the presence of hallucinations, although the exact type was not clarified, for which a prevalence of 27% (95% CI: 0.14-0.49) was calculated. Somatic, olfactory, and gustatory hallucinations were mentioned in only one study (56)each at rates of 41%, 2%, and 2%, respectively **Figure 3**.

We now examine the results on deaths by suicide and amphetamine use disorder.

#### 3.4.3 Suicidality

**Figure 4:**
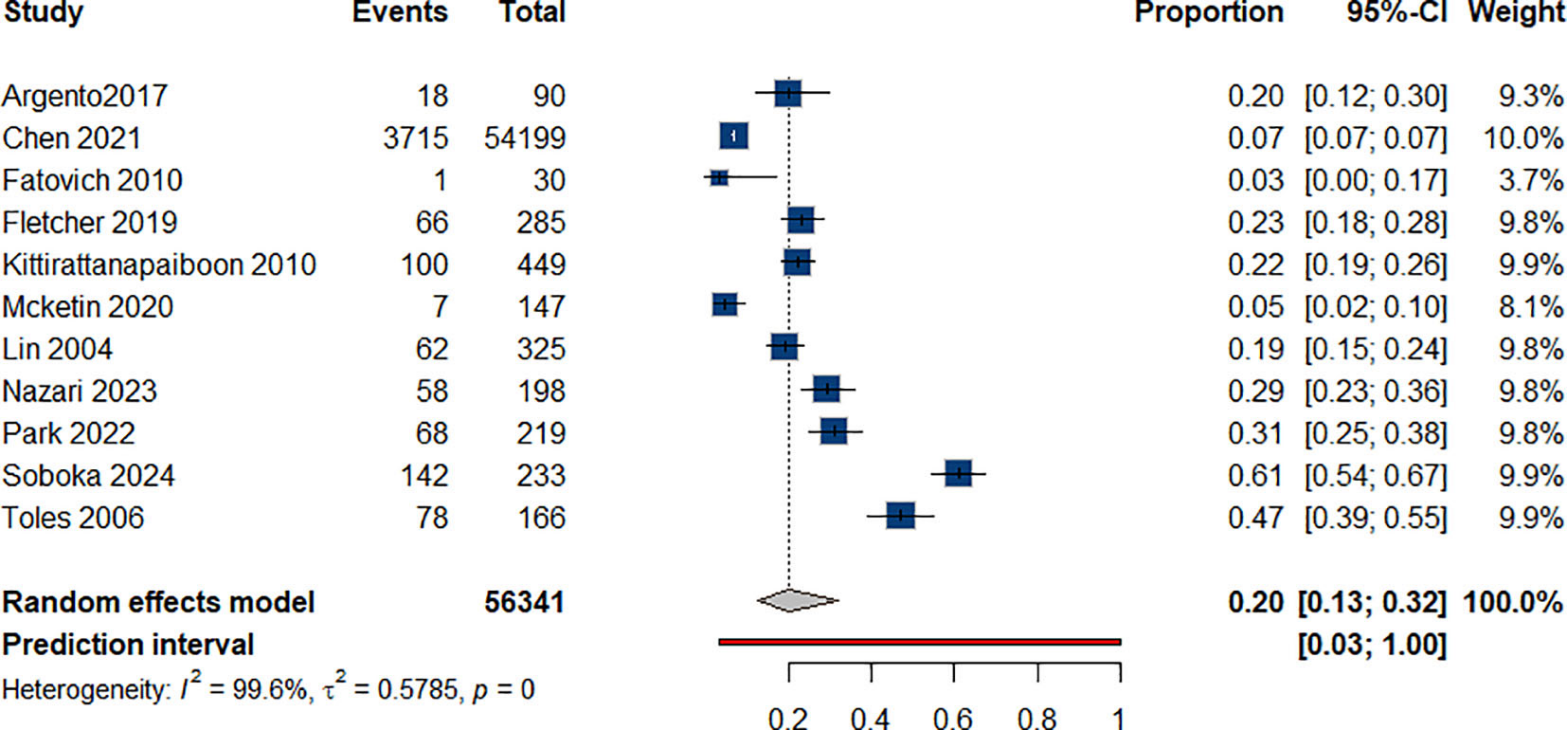
Prevalence of suicidality among individual with ATS use.

As reported in 11 studies (37, 39, 42, 46, 47, 56, 59–63) without clear delineation among different forms of suicidality, we estimated the rate of deaths by suicide to be 20% (95% CI: 0.13-0.32) among individuals with ATS use **Figure 4**.

#### 3.4.4 Suicidal ideation

**Figure 5:**
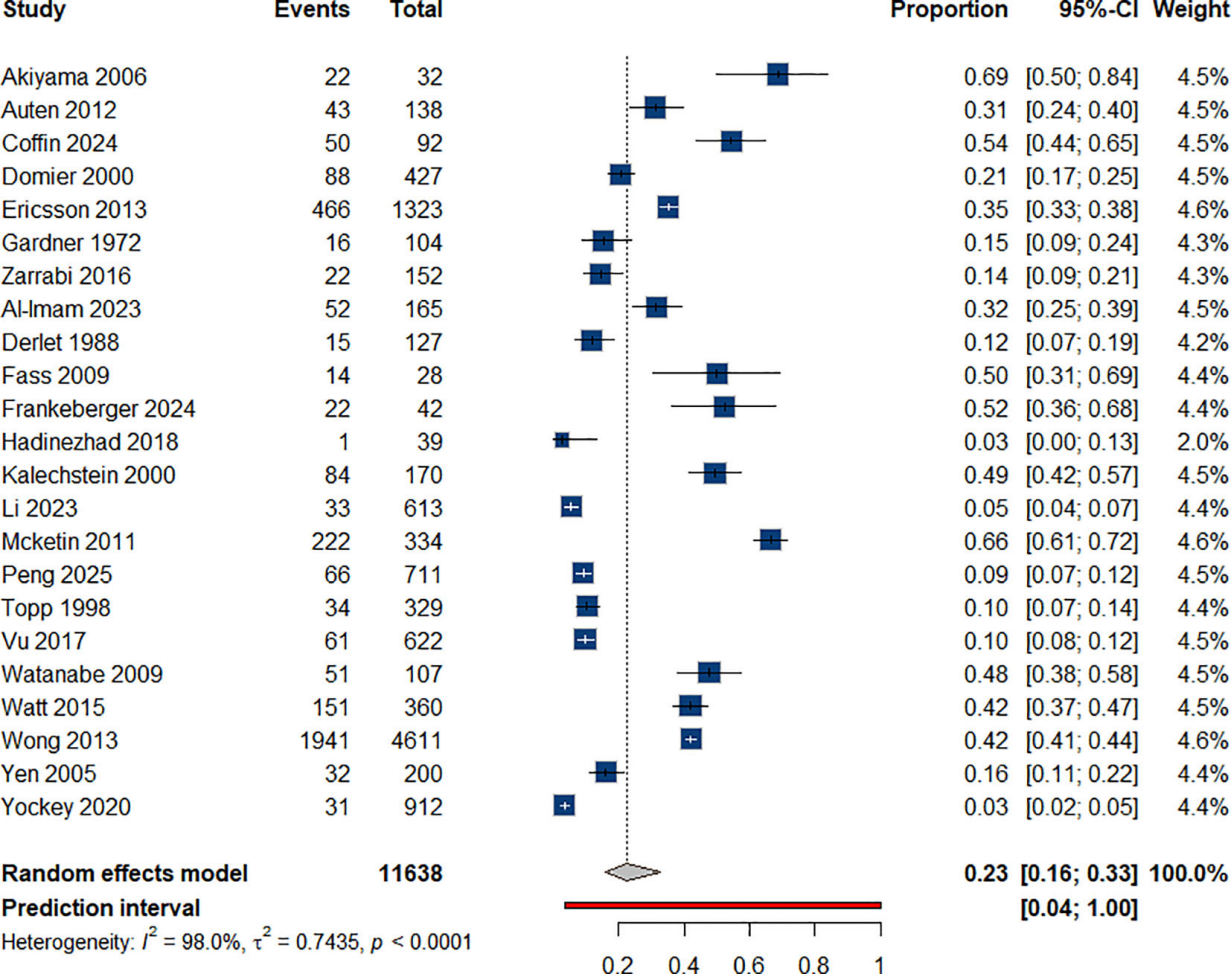
Prevalence of suicidal ideation among individual with ATS use.

We amassed data from 22 studies on suicidal ideation among individuals who use ATS (34–36) (38, 41, 44, 45, 48, 49, 51–55, 57, 64–70); accordingly, we estimated the prevalence rate to be 23% (95% CI: 0.16-0.33), **Figure 5**. For each year increase in the duration of amphetamine use, the odds of experiencing suicidal ideation increase by 21% (OR = 1.21). This effect was statistically significant, **Table 1**.

#### 3.4.5 Suicide attempts

**Figure 6:**
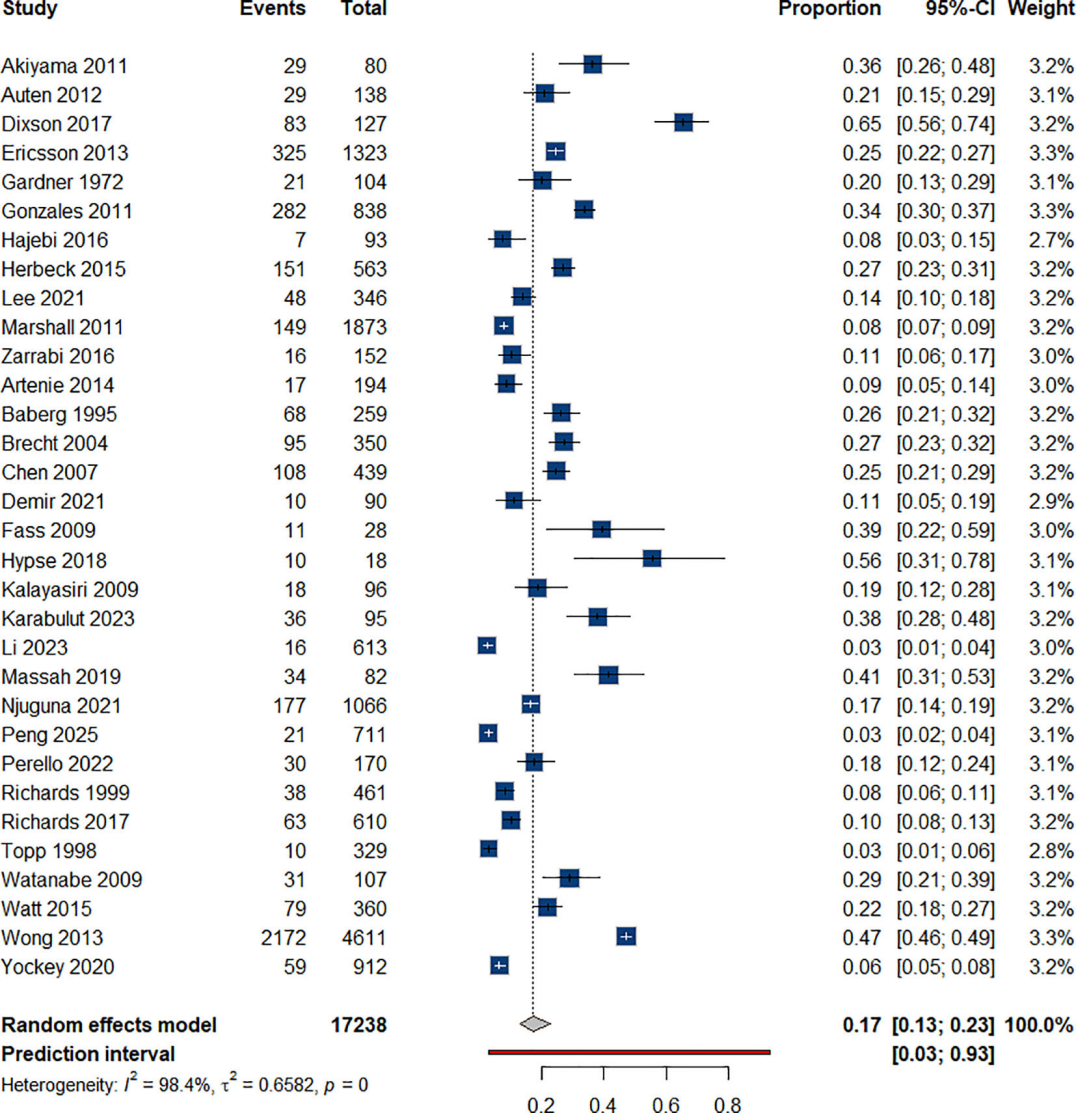
Suicide attempt rates among individual with ATS use.

Suicide attempts: Twenty-eight studies reported data on suicide attempts among individuals who use ATS (35, 39, 41, 43, 49, 51, 53, 57, 58, 65–67, 69–84). Our analysis revealed a prevalence rate of 17% (95% CI: 0.13-0.23). **Figure 6** presents the pooled prevalence estimate of 17% (95% CI: 0.13–0.23). For example, Richards et al. (2017) reported suicide attempt rates among methamphetamine users in emergency settings (75). The duration of ATS use did not have a statistically significant effect on the odds of attempting suicide, **Table 1**.

#### 3.4.6 Deaths by suicide

**Figure 7:**
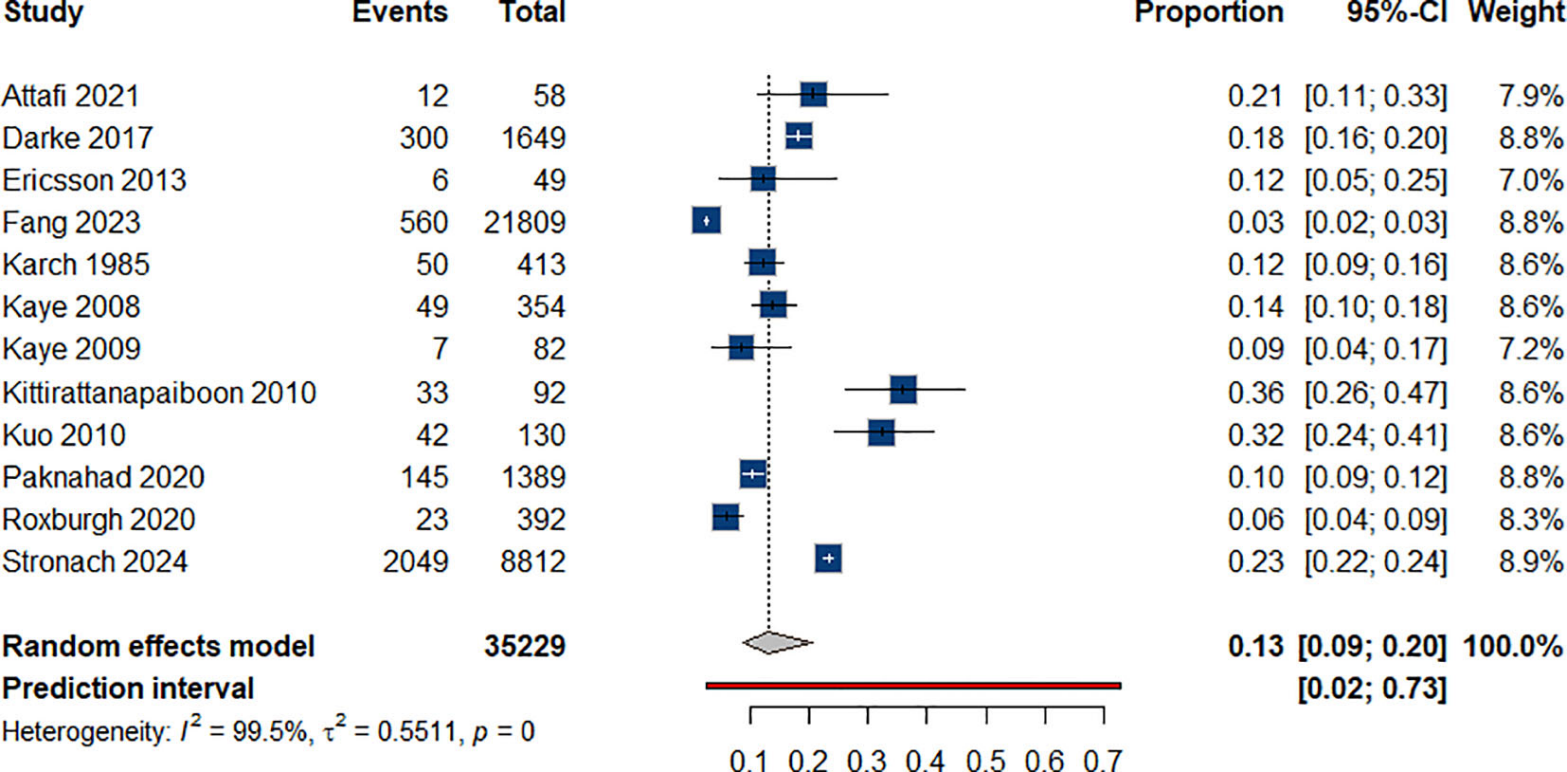
Rate of death by suicides among individual with ATS use.

We pooled data regarding the rate of successful deaths by suicide among individuals with ATS use from 12 studies (50, 56, 67) (85–93); the resulting estimated prevalence rate was 13% (95% CI: 0.09-0.20) **Figure 7**.

The pooled prevalence estimates stratified by attribution tier: **Table 2**. presents pooled suicide prevalence estimates categorized by attribution level for suicidal ideation. Tier 1 = 28.5%, Tier 2 = 22.6%, Tier 3 = 17.6%, For suicide attempts: Tier 1 = 20.6%, Tier 2 = 16.7%, Tier 3 = 12.9%. For complete suicide: Tier 1 = 16.2%, Tier 2 = 11.2%, Tier 3 = 8.0.Tier 3 = 8.0.

**Table 2:** Sensitivity analysis of prevalence estimates by study attribution strength.

Outcome	N (All Studies)	Prevalence (All)	N (Tier 1 Only)	Prevalence (Tier 1 Only)	N (Tiers 1 & 2)	Prevalence (Tiers 1 & 2)	Change in Prevalence (T1&2 vs All)
**Depression**	21	25.5%	3	31.7%	21	25.5%	0.0pp
**Suicidal Ideation**	23	22.6%	4	28.5%	18	23.9%	+1.4pp
**Suicide Attempts**	32	16.2%	5	20.6%	23	17.5%	+1.3pp
**Completed Suicide**	12	13.4%	6	16.2%	12	13.4%	0.0pp

**N** represents the number of studies included in that analysis subset.

**Prevalence** is the pooled prevalence estimate from the meta-analysis subset.

**Tier 1 Studies** are those with explicit temporal/causal attribution between ATS use and the outcome.

**Tier 2 Studies** are those with inferred attribution (confirmed ATS use disorder).

**Change** indicates the difference in percentage points (pp) between the Prevalence (Tiers 1 & 2) and the Prevalence (All Studies).

#### 3.4.7 Polysubstance use

**Figure 8:**
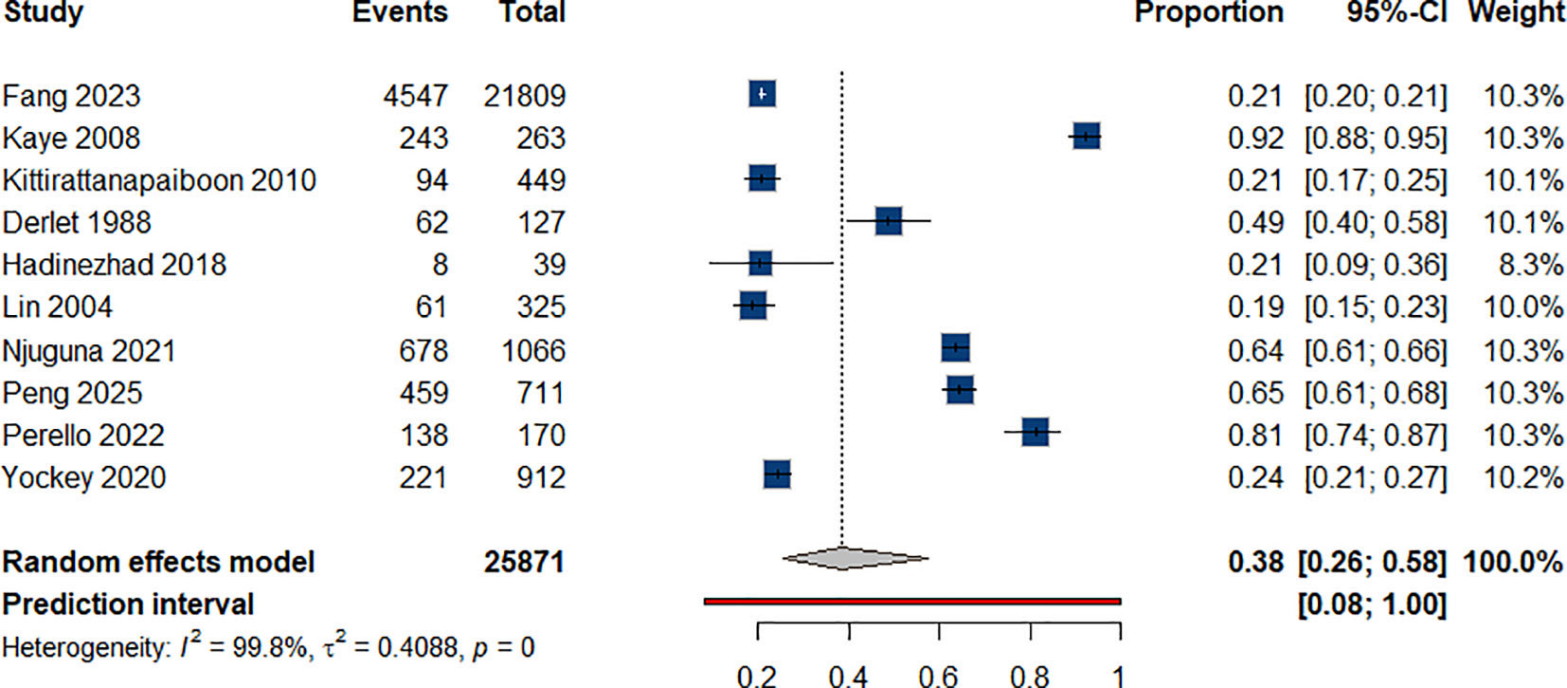
Percentage of polysubstance use among individual with ATS use.

Regarding polysubstance use, based on 10 (37, 42, 50, 54, 57, 64, 79, 81, 83, 86). We estimated that 38% of individuals who use ATS also used other substances **Figure 8**.

#### 3.4.8 Duration of ATS use

**Figure 9:**
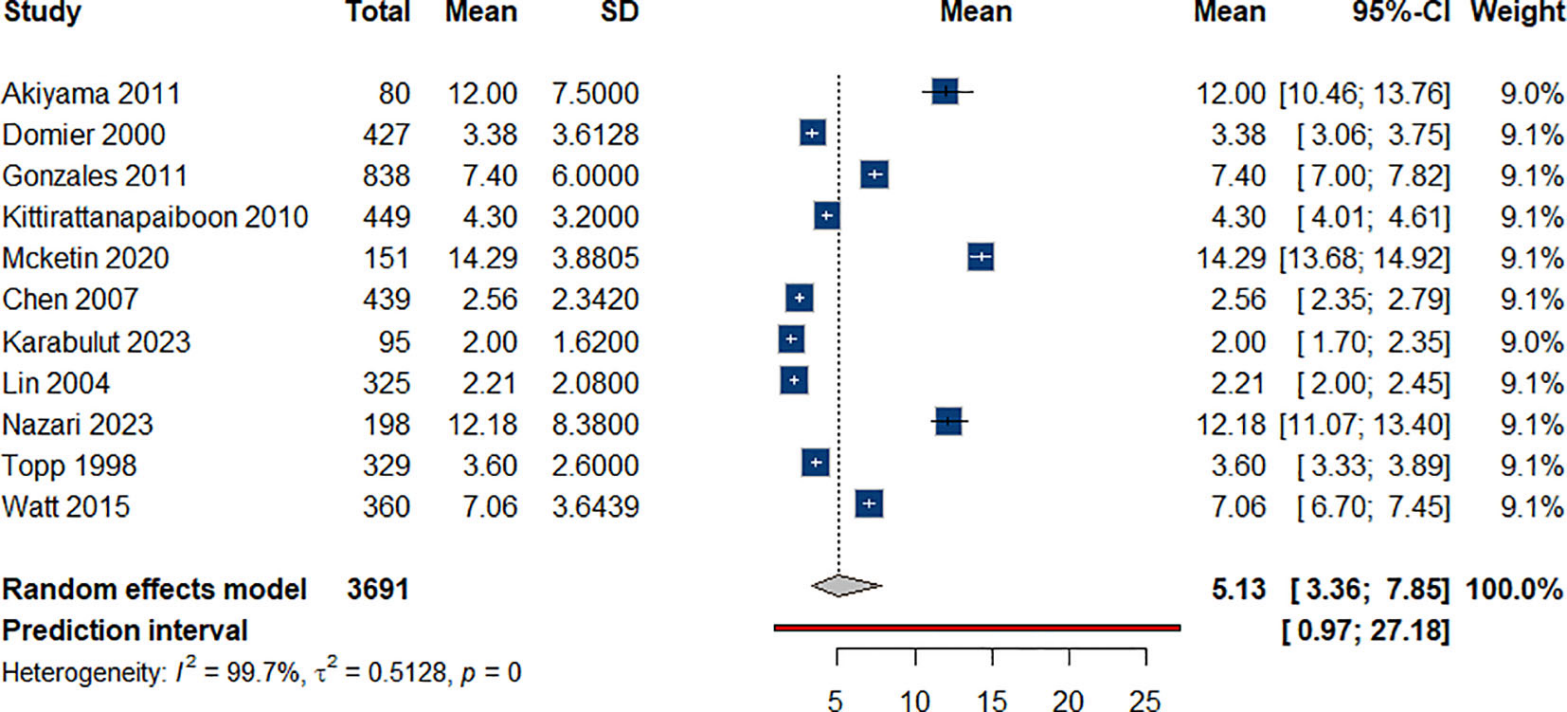
Mean duration of ATS use among individual with ATS use.

As for the duration of ATS use, the mean duration of ATS use among individuals who use ATS was approximately 5.13 years (95% CI: 3.36-7.85), as pooled from 10 studies (36, 37, 39, 42, 47, 51, 56, 68, 70, 73) **Figure 9**.

## 4 Discussion

Our findings confirm a substantial prevalence of suicidality among individuals who use ATS, including suicidal ideation (23%), attempts (17%), and deaths by suicide (13%). However, these estimates are based solely on ATS-using populations and do not include comparisons with the general population. A critical consideration in interpreting these findings is the absence of a comparison group (e.g., the general population or individuals with other substance use disorders). Consequently, the high prevalence rates reported here do not imply an excess or attributable risk specifically due to ATS use, but rather reflect the substantial burden of psychiatric comorbidity within this clinical population. This distinction is crucial, and our clinical implications are therefore focused on enhancing screening protocols and informing service-planning guidance rather than establishing a causal risk association.

The high degree of heterogeneity observed in the prevalence estimates (as indicated by the statistics in our forest plots) necessitates a contextual interpretation of the pooled results. This heterogeneity is likely driven by several key factors across the included studies: -1Clinical Setting and Sample Type: Prevalence rates varied significantly based on the recruitment setting. Studies from Emergency Department (ED) or inpatient psychiatric settings (often reflecting acute intoxication or withdrawal) typically reported higher prevalence of current suicidality compared to those from outpatient or community-based settings (which may capture more stable or chronic use). We interpret the pooled prevalence as a reflection of the overall burden across the spectrum of care, with the higher rates suggesting a critical need for screening in acute settings. -2Current vs. Lifetime Prevalence: The pooling of studies reporting both current (past 30 days or during hospitalization) and lifetime prevalence contributed to the observed variability. We have attempted to stratify these where possible, but the overall pooled estimate should be interpreted as a conservative measure of lifetime exposure to suicidal behavior within this population. -3 Assessment Tools and Diagnostic Rigor: Studies utilizing structured diagnostic interviews (e.g., SCID, MINI) or medical records for diagnosis of ATS use disorder and suicidality were pooled with those using screening tools (e.g., self-report questionnaires). The former generally provides a more rigorous, but potentially lower, prevalence estimate compared to the latter. This methodological difference is a major source of heterogeneity and underscores the need for standardized assessment in future research. -4 Polysubstance Use: The inclusion of studies with high rates of polysubstance use (Tier 3) introduces confounding, as the observed suicidality cannot be solely attributed to ATS. We have highlighted this in our limitations, but the heterogeneity in polysubstance use profiles (e.g., co-use with alcohol, opioids) further complicates the interpretation of the pooled prevalence. -5 Terminology and Diagnostic Criteria: The use of varying terminology across studies—specifically ‘use’ vs. ‘abuse’ vs. ‘use disorder’—reflects the evolution of diagnostic criteria (e.g., DSM-IV vs. DSM-5). We have standardized our reporting to ‘ATS use disorder’ where possible, but the underlying differences in the severity of the included samples remain a source of heterogeneity. Similarly, the duration measures (e.g., years of use) were often self-reported and varied widely, contributing to the difficulty in drawing firm conclusions about temporal relationships.

The tiered sensitivity analysis demonstrates a consistent gradient (Tier 1 > Tier 2 > Tier 3) across suicidality outcomes, explicit temporality and better exposure definition (Tier 1) reduce misclassification and increase detection of ATS associated suicidality, while inferred or polysubstance contexts (Tier 2/Tier 3) dilute attribution through information bias and confounding. Given the high co involvement of opioids in stimulant related events and the established harms of polysubstance use, Tier 3 should be interpreted as a burden within mixed use settings rather than an ATS specific risk. Tier 1 studies often draw from emergency, inpatient, or acute psychiatric settings where suicidality is assessed concurrently with ATS intoxication/withdrawal; this improves detection sensitivity and minimizes recall error.

Individuals with amphetamine-type stimulant (ATS) use frequently exhibit multiple psychiatric symptoms. The review showed that 26% experienced depression and 22% reported hallucinations. Among those with hallucinations, auditory types were present in 34% of cases, while visual types occurred in 32%. Importantly, individuals with psychotic symptoms often displayed more than one form of hallucination, indicating significant overlap among hallucination subtypes.

Several studies have confirmed the association between amphetamine use and a range of psychiatric disturbances, including depressive symptoms, violent behavior, impulsivity, and aggression (19, 34). According to Fang et al., individuals who use methamphetamine with a concurrent psychiatric disorder were significantly more likely to die by suicide (OR = 2.21; 95% CI: 1.84-2.64), with bipolar disorder showing the strongest association (OR = 2.25; 95% CI: 1.88-2.69) (50).

Rawson et al. reported that individuals using methamphetamine who experienced auditory hallucinations during treatment had a 60% dropout rate, compared with 40% among those without hallucinations (p < 0.05) (94). A strong link between suicide and depression or anxiety was also observed: Artenie et al. found that 45.1% of individuals using amphetamines who attempted suicide had received treatment for depression or anxiety, compared with 25.2% without such treatment (p<0.001) (95). Moreover, a history of any psychiatric disorder significantly increased the likelihood of suicide attempts among individuals using amphetamines (52.9% vs. 26.7%, p < 0.001). Further analysis revealed that treatment for anxiety or depression and a history of psychiatric disorders were associated with a 2.56-fold and 3.25-fold increase in suicide risk among individuals using methamphetamine, respectively (OR = 2.56; 95% CI: 1.84-3.56; p < 0.01 and OR = 3.25; 95% CI: 2.39-4.41; p < 0.01) (49). Methamphetamine-related neurobiological changes are thought to contribute to the persistence of psychiatric disturbances. Evidence suggests that stimulant exposure can induce alterations in brain function that manifest as psychotic symptoms, cognitive decline, and personality changes (37). These mechanisms provide a plausible context for the high rates of suicidality observed among individuals who use ATS, but they should be interpreted as supportive background rather than causal proof. In line with the aims of this meta-analysis, the emphasis remains on quantifying suicidality outcomes and their clinical implications, with biological mechanisms serving only as a concise explanatory framework (96).

Concurrent use of multiple substances was highly prevalent among individuals who use ATS disorder, with an estimated rate of 38%. Attafi et al. reported a significant association between concurrent use of multiple substances and an increased risk of suicide (90). Similarly, Ali et al. found that methamphetamine use, when combined with other substances, heightened the likelihood of impulsive and aggressive behaviors compared to single-drug use or use of non-methamphetamine substances (19). Rawson et al. further demonstrated that individuals enrolled in treatment programs for amphetamine use disorder were more likely to discontinue treatment if they concurrently used alcohol (56.8% vs. 43.2%, p < 0.05) (94).

In our analysis of ATS-associated suicidality, 20% of individuals exhibited suicidality, including ideation and attempts, while 23% reported suicidal thoughts, 17% attempted suicide, and 13% died by suicide. The estimated average duration of amphetamine use was 5.13 years.

Research has described myriad psychological, demographic, and social factors that contribute to ATS use disorder and, consequently, suicidality. Frankeberger et al. found that patients with a low educational status were more likely to develop amphetamine use disorder (38.1% vs. 28.6%, OR 5.03; p<0.05) (97). Lee et al. demonstrated that educational levels lower than high school were correlated with a risk of suicide among individuals with amphetamine use disorder (OR 2.83, p<0.05) (80).

Further, Rawson et al. showed that difficulties in school were associated with a reduced probability of completing methamphetamine treatment programs (50.4% vs. 49.6%, p<0.05) (94). Argento et al. established that higher educational attainment decreased the probability of suicide among people who use amphetamines (32.3% vs. 54.8%, p=0.017) (60).

Adverse childhood experiences (ACEs) are among the most prominent factors associated with increased risk of suicide. Lee et al. observed that individuals who had two or more ACEs were 3.29 times more likely to die by suicide, and those with three or more ACEs faced an even greater risk, 5.39 times higher (p<0.05). They analyzed the relationship between amphetamine and ACEs and found that amphetamine acts as a mediator between ACEs and suicide among individuals with substance use disorder (70). Argento et al. showed that among individuals who use amphetamines, childhood trauma was a prominent predictor of suicidality, as those who displayed suicidality experienced childhood trauma at a significantly higher rate than those who were not suicidal (77.4% vs. 43.2%, p < 0.001) (60).

Gender differences in suicidality among individuals who use methamphetamine reveal distinct patterns. Kaye et al. reported that male individuals with methamphetamine use disorder were more likely to die by suicide than females (14% vs. 12%) (86). A finding echoed by Ericsson et al., who noted that 12% of methamphetamine-related deaths among men were due to suicide (67). Conversely, Auten et al. observed a female predominance in suicidal ideation (65%) and suicide attempts (66%) among individuals using methamphetamine (66). Similarly, Artenie et al. identified male gender as a protective factor against substance use-related suicide deaths (HR = 0.47, 95% CI: 0.30-0.74, p < 0.05), while female individuals demonstrated a higher likelihood of attempting suicide (26.8% vs. 15.7%, p = 0.014) (49). Supporting this trend, Lee et al. found that female individuals using amphetamines were significantly more likely to die by suicide (OR = 3.14; p<0.05) (80).

Frankeberger et al. revealed that methamphetamine use disorder was more common among people with an insecure housing status (42.9% vs. 12.6%, p<0.001) (97). Dixson et al. found that individuals who moved between three or more places of residence within six months were more likely to use methamphetamine (52% vs. 38.2%, p=0.025), and those with a generally unstable housing status were also more likely to use methamphetamine (27.6% vs. 15%, p=0.012) (76). Artenie et al. reported that sex work among individuals using amphetamines was highly predictive of suicide (OR 2.06, 95% CI: 1.26-3.38, p<0.01), as 23.9% of individuals with amphetamine use disorder who engaged in sex work within six months of the study displayed suicidality, compared with 10.7% of those who did not (p<0.001) (95). Moreover, Paydar et al. showed that suicidality was significantly predictive of treatment outcomes in individuals using methamphetamine, with those who had a history of suicidality displaying considerably worse treatment outcomes (OR 30.33, 95% CI: 3.11-295.24, p=0.003) (98). Additionally, Zarrabi et al. underscored the link between suicidal behaviors in individuals using methamphetamine and amphetamine-induced aggression, which is partially directed toward the self (53).

## 5 Conclusion

This review highlights the high prevalence of suicidality and psychiatric comorbidities among individuals who use ATS, including depression and hallucinations, bipolar disorder, violent behavior, impulsivity, and aggression. Sociodemographic factors, including low education levels, ACEs, unstable housing, and gender, also affect suicidality. While some studies report higher male mortality from suicide, others highlight a female preponderance in suicidality, warranting further sex-specific investigations. These findings underscore the complexity of ATS-related mental health challenges and suggest that targeted interventions for this population may be warranted. However, given the absence of comparative data with individuals without ATS use, these recommendations should be considered preliminary. Future research should focus on comparative analyses and longitudinal studies to better inform prevention strategies.

### 5.1 Limitations

A critical limitation is the variability in how studies established the temporal and causal relationship between ATS use and suicidality. While some studies explicitly documented suicidality during active intoxication or withdrawal, others reported lifetime prevalence among individuals who use ATS disorder without clarifying timing. This methodological heterogeneity limits our ability to distinguish ATS-attributable suicidality from suicidality due to pre-existing psychiatric disorders or psychosocial factors. Future research should employ standardized assessment tools that capture the temporal sequence of substance use and suicidality.

The over-inclusion of cross-sectional studies in the current literature represents a significant limitation to the temporal interpretation of our findings. Specifically, the cross-sectional design prevents us from establishing whether the duration of ATS use is a preceding risk factor for suicidality or if the two are concurrent manifestations of a shared underlying vulnerability. This highlights the urgent need for prospective, longitudinal studies that can track individuals over time to clarify the causal and temporal pathways between ATS use, psychiatric comorbidity, and the development of suicidal ideation and attempts. Future meta-analyses should employ case-control designs or comparative cohort studies to establish whether ATS use confers incremental suicide risk beyond baseline rates in matched populations. Such research is essential to justify resource allocation for ATS-specific suicide prevention programs.

Further, only papers written in English were included, and the included studies were highly heterogeneous, with diverse research methodologies and some cross-sectional research.

## Data Availability

The original contributions presented in the study are included in the article/**Supplementary Material**. Further inquiries can be directed to the corresponding author.

## Supplementary material

SUPPLEMENTARY TABLE 1 Data collection.

SUPPLEMENTARY TABLE 2 Sensitivity analysis.

SUPPLEMENTARY TABLE 3 Summary and baseline characteristics of the included studies.

SUPPLEMENTARY TABLE 4 Risk of bias for cross-sectional and cohort studies.
