# Supplementary material for: Suicide and psychiatric disorders associated with amphetamine type stimulant use: a systematic review and meta-analysis
Source: Front Psychiatry. 2026 Mar 13;17:1654091. doi: 10.3389/fpsyt.2026.1654091 (PMC13022986; doi:10.3389/fpsyt.2026.1654091)
Supplement: SUPPLEMENTARY TABLE 3 — Summary and baseline characteristics of the included studies. [file Table3.docx]

**Supplementary Table 3:** Summary and baseline characteristics of the included studies.

| **Study ID** | **Design** | **Country** | **Substance/Group** | **No. of patients** | **No. of METH/AMPH/DMDA/ECSTASY users** | **Additional substance** | **Female no. (%)** | **Age at first consultation (y)** | | **Age at the onset of substance abuse (y)** | | **Abuse period (y)** | | **Latencies from beginning of substance abuse to emergence of an initial psychotic episode (y)** | | **Education** | **Marital status** | **Employment** | **Route** | **Ethnicity** | **Conclusion** |
| --- | --- | --- | --- | --- | --- | --- | --- | --- | --- | --- | --- | --- | --- | --- | --- | --- | --- | --- | --- | --- | --- |
| Akiyama 2006 | Prospective Cohort | Japan | Meth | 32 | 1. Meth: 32 (100) | NR | 32 (100) | 31.7 | 5.7 | NR | NR | 19:12 | 5.6 | 4.6 | 3.7 | NR | NR | NR | NR | NR | "The study concludes that both psychotic and affective symptoms significantly influence the therapeutic response and prognosis of methamphetamine (METH) psychosis in female patients. It identifies three distinct groups of patients based on the severity of their symptoms, which exhibited different prognoses during pharmacological treatment. The average degree of extrapyramidal symptoms correlated with the daily dose of antipsychotics and the length of treatment required for symptom improvement. The findings suggest that long-term METH abuse contributes to persistent psychotic states, which may be refractory to treatment." |
| Akiyama 2011 | Prospective Cohort | Japan | Meth | 80 |  | NR | 80 (100) | 32.7 | 7.5 | 19.7 | 5.1 | 12 | 7.5 | 4.1 | 4.9 | NR | NR | NR | 1. Intravenous: 72 (90) 2. Intravenous+smoking: 6 (7.5) 3. Smoking only: 2 (2.5) |  | "The study indicates that psychiatric symptoms did not differ significantly between patients with spontaneous recurrence and those with persistent METH psychosis. A notable presence of premorbid psychiatric disorders was found in 24% of subjects, suggesting a spectrum of drug-associated psychiatric disorders. The findings highlight the need for further research on the long-term trajectory of METH psychosis due to its psychiatric sequelae. Subjects with spontaneous recurrence had longer abstinence periods and less frequent premorbid psychiatric disorders compared to those with protracted psychosis. The study emphasizes the complexity of METH psychosis, influenced by both drug exposure and individual psychiatric history." |
| Argento 2017 | Prospective Cohort | Canada | Suicidality | 31 | 1. Crystal Meth: 18 (58.1) 2. MDMA/ecstasy: 59/290 (20.34) | 1. Psychedelic 2. Cannabis 3. Opioid  4. Crack 5. Cocaine 6. Heroin | 31 (100) | 36.1377 | 15.5437 | NR | NR | NR | NR | NR | NR | 1. High school or greater: 10 (32.3) |  |  | 1. Non-injection drug use: 27 (87.1)  2. Injection drug use: 16 (51.6) |  | "The study highlights a critical public health concern due to high suicidality rates among marginalized women. Naturalistic psychedelic use is associated with reduced suicidality among sex workers. Other drug use and childhood trauma increase the risk of suicidality. This research is the first to longitudinally explore the link between psychedelic use and suicidality. Findings support further investigation into the therapeutic potential of psychedelics for mental health." |
|  |  |  | No suicidality | 259 | 1. Crystal Meth: 72 (27.8) |  | 259 (100) | 35.6492 | 9.6913 | NR | NR | NR | NR | NR | NR | 1. High school or greater: 142 (54.8) |  |  | 1. Non-injection drug use: 148 (57.1)  2. Injection drug use: 81 (31.3) |  |  |
| Attafi 2021 | Retrospective cohort | Saudi Arabia | AMPH Related death | 58 | 1. AMPH: 58 (100) | 1. Cathinone 2. Cathine 3. THC 4. Methamphetamine 5. Ethanol | NR | 31.9 | 8.2 | NR | NR | NR | NR | NR | NR | NR | NR | NR | NR | NR | "The study highlights a rising trend in fatalities linked to amphetamines combined with ethanol, THC, and cathinone. Higher fatalities involving amphetamines correlate with increased suicidality and homicide rates, especially in individuals under 35. Further research is necessary to explore the relationship between tissue amphetamine concentrations and death manner. Polydrug use, particularly with amphetamines, is a significant risk factor for fatal overdoses. The findings emphasize the need for increased awareness among healthcare providers regarding these fatalities." |
| Auten 2012 | Retrospective cohort | USA |  | 293 | 1. Meth: 293 (100) | NR | 167 (57) | 15.6358 | 1.2246 | NR | NR | NR | NR | NR | NR | NR | NR | NR | 1. Inhalation: 226 (77)  2. Ingestion: 64 (22)  3. Injection: 3 (1) |  | "The study highlights common suicidal ideation and attempts among adolescents abusing methamphetamines. Methamphetamine abuse poses risks for both suicidal and nonsuicidal users in emergency settings. Vigilance in screening for suicidality is essential for healthcare providers. Increased rates of suicidal ideation and attempts were found compared to population surveys. The findings align with previous literature indicating high rates of suicidal ideation in methamphetamine-abusing adolescents. A significant percentage of cases involved suicide attempts, emphasizing the need for intervention. The paper underscores the importance of a multidisciplinary approach to care for affected youth. Overall, methamphetamine abuse is linked to heightened risks of depression and suicidal behavior." |
| Chen 2021 | Retrospective cohort | USA | AMPH | 54199 | 1. AMPH: 54199 (100) | 1. Marijuana 2. Cocaine 3. Amphetamines & related compounds 4. Heroin 5. Oxycodone (± combinations) 6. Hydrocodone (± combinations) 7. Ethanol 8. Benzodiazepines | 18,100 (33.8) | 29.3 | 12.9 | NR | NR | NR | NR | NR | NR | NR | NR | NR | NR | NR | "The rate of exposure to methamphetamine in the US initially declined after the Combat Methamphetamine Epidemic Act of 2005. However, since 2007, both the rate and severity of methamphetamine exposures have significantly increased, particularly among individuals aged 20 years and older. There is a pressing need for enhanced prevention efforts, which should include coordinated public policy to limit access to methamphetamine, prevention of initiation among adolescents and young adults, and improved access to treatment for co-occurring mental health and substance use disorders. Additionally, targeted educational initiatives are necessary to address the risks of unintentional exposures among children." |
| Coffin 2024 | Retrospective cohort | USA |  | 92 | 1. Meth: 92 (100) | NR | 18 (19.5) | 40 | 9.8 | NR | NR | NR | NR | NR | NR | NR | NR | NR | NR | 1. Hispanic, Latine, or Spanish origin: 17 (18) 2. Not of Hispanic, Latinx, or Spanish origin: 73 (79) 3. Unknown/Chose not to disclose: 2 (2) | "There is no established intervention for self-management of acute psychiatric symptoms from methamphetamine use. Methamphetamine Assist Packs led to significantly fewer psychiatric emergency visits at two and six months post-receipt. Further research, including randomized controlled trials, is needed to confirm these preliminary findings. The intervention may reduce adverse events related to methamphetamine-induced psychosis. The study supports further implementation and evaluation of Methamphetamine Assist Packs as a public health intervention. The hypothesized benefits may require greater availability and pairing with counseling for sustained effects." |
| Darke 2017 | Retrospective cohort | Australia | Meth related death | 1649 | 1. Meth: 1649 (100) | 1. Amphetamine 2. Methamphetamine 3. Cocaine 4. MDMA 5. MDA 6. Hypnosedatives 7. Diazepam 8. Temazepam 9. Alprazolam 10. Oxazepam 11. Clonazepam 12. Nitrazepam 13. Flunitrazepam 14. Pregabalin 15. Zolpidem 16. Alcohol | 357 (21.6) | 36.9 | 9.7 | NR | NR | NR | NR | NR | NR | NR | 1. Married: 477 (28.9)  2. Not married: 1097 (66.5)   3. Unknown: 75 (4.5) | 1. Employed: 554 (33.6)  2. Not employed: 1009 (61.2)  3. Unknown: 86 (5.2) | 1. Injection: 920 (55.8) |  | "The study concluded that methamphetamine death rates in Australia doubled from 2009 to 2015, indicating a significant public health issue. Accidental drug toxicity was the most frequent cause of death, while natural disease, suicide, and accidents accounted for more than half of the fatalities. The majority of cases involved polydrug use, with opioids and hypnosedatives being the most commonly detected substances alongside methamphetamine. The findings highlighted the need for increased treatment options for methamphetamine users and greater awareness of the associated health risks, particularly regarding cardiovascular disease and suicide." |
| Darke 2018 | Retrospective cohort | Australia | Meth related death | 300 | 1. Meth: 300 (100) | 1. Amphetamine 2. Alcohol 3. Cannabis (D9 THC) 4. Hypnosedatives 5. Diazepam 6. Temazepam 7. Oxazepam 8. Alprazolam 9. Clonazepam 10. Nitrazepam 11. Flunitrazepam 12. Pregabalin 13. Zolpidem 14. Antidepressants 15. SSRIs 16. Tetracyclics 17. SNRIs 18. Tricyclics 19. MAOIs 20. Opioids 21. Morphine 22. Methadone 23. Oxycodone 24. Tramadol 25. Fentanyl 26. Antipsychotics 27. Quetiapine 28. Olanzapine 29. Clozapine 30. Risperidone 31. Flupenthixol 32. Zuclopenthixol 33. Paliperidone 34. Other psychostimulants 35. MDMA 36. Cocaine 37. Mephedrone | 69 (23) | 33.1 | 8.5 | NR | NR | NR | NR | NR | NR | NR | 1. Married/de facto relationship: 98 (32.7) | 1. Employed: 125 (41.8) | 1. Injection: 78 (26.0) |  | "Methamphetamine-related suicide represents a significant public health issue in both metropolitan and rural areas of Australia. The study found that methamphetamine-related suicide rates tripled during the study period. The majority of cases involved violent methods, particularly hanging, which was used in 70% of cases. A notable proportion of cases had a history of previous suicide attempts and psychosis. Recent life events, such as relationship breakups, were significant factors preceding suicides." |
| Dixson 2018 | Cross sectional | Australia | Meth | 127 | 1. Meth: 127 (100) 2. Ecstasy: 56 (44.1) | 1. Cannabis 2. Alcohol 3. Tobacco 4. Opioids 5. Ecstasy and related drugs 6. Cocaine 7. Hallucinogens 8. Other | 127 (100) | 16.48 | 0.8308 | NR | NR | NR | NR | NR | NR | NR | NR | NR | NR | NR | "The study highlights the significant role of trauma in adolescent females using methamphetamine in Australia. Methamphetamine users showed higher rates of unstable accommodation and suicide attempts compared to non-users. Trauma-informed care is essential for addressing the complex needs of these adolescents. The introduction of a standardized trauma inventory is recommended for better assessment. Qualitative findings emphasize the need for tailored treatment approaches for methamphetamine users. Increased attention to preventing trauma and violence in youth is crucial for effective treatment." |
| Domier 2000 | Cross sectional | USA | Meth | 427 | 1. Meth: 427 (100) | NR | NR | NR | NR | NR | NR | 3.3846y | 3.6128 | NR | NR | NR | NR | 1. Unemployed: 265 (62) | 1. Injection: 55 (12.9) 2. Sniffed: 338 (79)  3. Smoked: 104 (24.3) 4. Ingested orally: 63 (14.75) | 1. White: 349 (81.7)  2. Hispanic and other: 67 (15.8)  3. African American: 6 (1.3) | "The research concludes that there are significant differences between injecting and non-injecting methamphetamine users in terms of medical, psychological, and criminal histories. Injectors reported higher incidences of psychotic states, including hallucinations and suicidal ideation, compared to non-injectors. Injectors also experienced more severe depression and reported unique medical concerns, such as sexual dysfunction and a higher frequency of HIV positivity. Understanding these differences is crucial for tailoring treatment approaches and improving medical care for injecting users, potentially influencing future research on drug addiction." |
| Ericsson 2014 | Retrospective cohort | Sweden | AMPH | 1396 | 1. AMPH: 1396 (100) | 1. Alcohol 2. Heroin 3. Other opioids 4. Sedatives 5. Cocaine 6. Amphetamine 7. Cannabis 8. Tobacco | 209 (15) | 37.4 | 6.774 | NR | NR | 10.1893Y | 6.3224 | NR | NR | NR | NR | NR | NR | NR | "The study concludes that primary amphetamine users in the Swedish criminal justice system experience significant excess mortality, predominantly due to violent and drug-related causes, which are potentially preventable. It identifies that mortality is more closely associated with the use of non-stimulant drugs, such as sedatives, rather than the frequency of amphetamine use itself. The findings highlight the need for targeted interventions and further research to understand the risk factors contributing to the high mortality rates in this population. The study emphasizes the importance of addressing polydrug use in treatment strategies for amphetamine users." |
| Fang 2023 | Retrospective cohort | Taiwan | Meth With a psychiatric disorder | 10,425 | 1. Methamphetamine only: 8067 (77.38)  2. Combined with other substance use disorder: 2358 (22.62) | NR | 2572 (24.67) | 33.21 | 9.12 | NR | NR | NR | NR | NR | NR | NR | NR | NR | NR | NR | "The study concluded that co-occurring psychotic disorders significantly contribute to increased mortality and suicide rates among patients with methamphetamine use disorder (MUD). It was found that patients with MUD and any psychiatric disorder had a higher risk of all-cause mortality (adjusted hazard ratio: 1.6) and suicide (adjusted hazard ratio: 2.27) compared to those without psychiatric disorders. The findings emphasize the need for clinicians to provide appropriate treatment and timely interventions for individuals with MUD and co-occurring mental disorders to mitigate these risks." |
|  |  |  | Meth Without a psychiatric disorder | 11,384 | 1. Methamphetamine only: 9195 (80.77) 2. Combined with other substance use disorder: 2189 (19.23) | NR | 1641 (14.41) | 31.58 | 9.93 | NR | NR | NR | NR | NR | NR | NR | NR | NR | NR | NR |  |
| Fatovich 2010 | Prospective Cohort | Australia | Polysub. | 30 | 1. Crystal methamphetamine: 16 (53) 2. Amphetamine: 9 (30) 3. Ecstasy: 23 (76) | 1. Crystal methamphetamine  2. Amphetamine  3. Ecstasy  4. Marijuana  5. Alcohol 6. Cocaine 7. Heroine 8. LSD | 11 (36.6) | 26.7 | 5.4 | 18 | 3.1 | 7.5 | 6.4219 | NR | NR | NR | NR | NR | 1. IV 2. Oral 3. Intranasal |  | "The study found a prevalence of clinically occult cerebral abnormalities in active amphetamine users presenting to the emergency department. One in five patients exhibited abnormalities on MRI scans, primarily unidentified bright objects (UBOs). Most UBOs were located in the frontal region, consistent with previous findings in methamphetamine users. The research suggests a potential link between amphetamine use and cognitive decline or increased stroke risk. Limitations include a small sample size and the challenge of attributing causation due to poly-drug use. The findings highlight serious long-term effects of amphetamine use, including mood and memory disturbances." |
| Fletcher 2018 | Cross-Sectional | USA | Meth | 285 | 1. Meth: 285 (100) | 1. Marijuana use disorder 2. Alcohol use disorder 3. Cocaine use disorder 4. Inhalants use disorder 5. Opiate use disorder 6. Hallucinogen use disorder 7. Non-opiate sedative use disorder 8. Amphetamine-type stimulant use disorder 9. PCP use disorder | 0 (0) | 42 | 11 | NR | NR | NR | NR | NR | NR | NR | NR | NR |  | 1. African American/Black: 125 (44) 2. Hispanic/Latino: 71 (25) 3. Other/Unknown: 89 (31) | "The study indicates elevated rates of mental health and substance use disorders among methamphetamine-using MSM compared to non-MSM males. Methamphetamine use is linked to neuronal damage and mental health disorders. Significant associations were found between methamphetamine use disorder severity and various mental health disorders. The report provides important estimates of DSM-5 mental health disorders and substance use severities among methamphetamine-using MSM. Mental health counseling may be necessary for those seeking treatment for methamphetamine use due to elevated disorder rates. The study's limitations include restricted generalizability and limited statistical power for low-prevalence disorders." |
| Gardner 1972 | Prospective Cohort | UK | AMPH | 104 | 1. AMPH: 104 (100) | 1. Heroin 2. Methadone 3. Other opioid 4. Cocaine 5. Barbiturates 6. Non-barbiturate hypnotic 7. Amphetamines 8. Amphetamine-barbiturate pill 9. Hallucinogens (e.g. L.S.D.) 10. Cannabis | 21 (20) | 23.1 | 8.0565 | 18.6 | 6.3228 | 3.1 | 3.0847 | NR | NR | NR | NR | 1. Working full-time: 36 (34.6) 2. Working part-time: 5 (4.8) | 1. IV: 35 (33.6) 2. Oral: 65 (67.6) |  | "The paper reports findings on 104 users of non-opioid drugs, mainly amphetamines, attending a drug dependence clinic in London. It highlights that nearly one-third began amphetamine misuse while at school or shortly after. Amphetamine psychosis occurred in 35% of cases, more frequently with intravenous abuse. The paper suggests that 'maintenance therapy' is unlikely to be effective for amphetamine dependence. A strong case is made for compulsory notification of individuals dependent on amphetamines under the Misuse of Drugs Act." |
| Gonzales 2011 | Retrospective cohort | USA | Meth | 838 | 1. Meth: 838 (100) | NR | 428 (51.1) | 31.4 | 8.03 | NR | NR | NR | NR | NR | NR | 1. High school education or more: 651 (77.7) | 1. Never married: 418 (49.9) 2. Divorced/separated/widowed: 277 (33.1) 3. Married: 144 (17.2) | 1. Employed: 614 (73.3) |  | 1. White: 528 (63.0) 2. Hispanic: 151 (18.0) 3. Asian/Pacific Islander: 118 (14.1) 4. Native American/Alaskan Indian: 25 (3.0) 5. African American: 16 (1.9) | "The study expands the scholarly examination of quality of life (QOL) among methamphetamine (MA)-dependent users entering treatment, highlighting the lack of coherent profiles in existing literature. It identifies critical clinical determinants of QOL, such as drug severity behaviors, interpersonal dysfunction, and medical and psychiatric comorbidities, which should be targeted for change in treatment planning. Understanding these factors can help treatment providers identify risks and barriers that exacerbate substance dependence and worsen treatment outcomes. The findings emphasize the importance of routine health status monitoring in treatment programs for MA users." |
| Hajebi 2016 | Prospective Cohort | Iran | Meth induced psychosis | 55 | 1. Meth: 55 (100) | NR | 11 (20) | 31.44 | 7.34 | NR | NR | NR | NR | NR | NR | 1. Preliminary school: 9 (16.4)  2. High school: 41 (74.5) 3. College education: 5 (9.1) | 1. Never-married: 34 (61.8)  2. Currently married: 8 (14.5) 3. Separated/divorced: 13 (23.7) | 1. Employed: 7 (12.8) |  |  | "Methamphetamine-induced psychosis (MAP) does not have a favorable course; positive symptoms persist after six months, similar to non-affective psychosis (NAP). High rates of re-hospitalization and suicide attempts are observed in MAP patients. Future studies are needed to clarify whether enduring delusional beliefs are drug-induced or indicative of schizophrenia. The cause of chronic negative symptoms in MAP patients remains unclear, possibly due to neurological injury or schizophrenia progression. MAP is not self-remitting; many patients experience chronic courses and relapses requiring intensive follow-up. The study assessed 165 subjects, revealing significant differences in symptom trends among MAP and other psychotic disorders." |
| Herbeck 2015 | Prospective Cohort | USA | Meth | 563 | 1. Meth: 563 (100) | NR | 223 (39.6) | 32.5 | 8.7 | NR | NR | NR | NR | NR | NR | 1. Less than high school: 175 (31) 2. High school diploma: 135 (24) 3. Post-high school education: 259 (46) |  | 1. Employed: 203 (36)  2. Unemployed: 360 (64) |  | 1. African American: 97 (17.2) 2. Hispanic: 165 (29.3) 3. White: 239 (42.5) 4. Native American: 15 (2.7) 5. Other/multi-racial: 47 (8.3) | "The study examines mortality causes and health characteristics in methamphetamine users over 8-10 years, highlighting treatment status differences. Treated and untreated groups showed divergent health outcomes, informing public health strategies for both populations. Higher mortality rates were associated with anxiety and depression in the treated group, unlike the untreated group. Nearly half of the deceased participants had attempted suicide, indicating significant mental health issues. The study emphasizes the need for further research on long-term health consequences in methamphetamine-using populations." |
| Rawson 2005 | Retrospective cohort | USA |  | 305 | 1. Meth: 90 (29.5) | NR | 91 (30) | 16 | 0.8709 | NR | NR | NR | NR | NR | NR | NR | NR | NR | NR | 1. Caucasian: 169 (55.3) 2. Latino: 101 (33.1) 3. African American: 24 (8.0) 4. Asian: 9 (3.0) 5. Other: 3 (1.0) | "The study identified key factors influencing treatment response among methamphetamine-abusing adolescents, highlighting the need for tailored treatment strategies. Methamphetamine-using adolescents exhibited higher psychosocial dysfunction and substance use at treatment discharge compared to non-meth users. Suicidal ideation and substance use during treatment were significant predictors of early dropout. The findings suggest a necessity for enhanced treatment programming for methamphetamine-using adolescents to improve outcomes. The study contributes valuable insights into adolescent substance abuse, emphasizing the importance of addressing specific treatment needs." |
| Karch 1999 | Retrospective cohort | USA | Meth Unrelated death | 143 | 1. Meth: 413 (100) | NR | 61 (14.8) | 35 | 10.5 | NR | NR | NR | NR | NR | NR | NR | NR | NR | NR | 1. Caucasian: 307 (74.3) 2. African-American: 61 (14.7) 3. Asian: 17 (4.1) 4. Hispanic: 7 (1.7) | "The study suggests a strong association between methamphetamine use and various health issues, including cardiac enlargement and subarachnoid hemorrhage. Methamphetamine-related deaths are defined as those where the drug directly contributed to the decedent's death. The manner of death for methamphetamine users is often classified as accidental, complicating the understanding of its impact. The research highlights the aging population of drug users, with a mean age of 36.8 years among decedents. The paper emphasizes the need for better understanding of methamphetamine's pathology and toxicology." |
|  |  |  | Meth Related death | 270 |  | NR |  | 38 | 9.4 | NR | NR | NR | NR | NR | NR | NR | NR | NR | NR |  |  |
| Kaye 2008 | Retrospective cohort | Australia | Meth death | 371 | 1. Meth: 371 (100) | 1. Amphetamine 2. Benzodiazepines 3. Morphine 4. THC 5. Codeine 6. Antidepressants 7. Alcohol 8. Methadone 9. MDMA 10. MDA 11. Antipsychotics 12. Cocaine/benzoylecgonine 13. Ketamine | 86 (23.2) | 32.7 | 11.5 | NR | NR | NR | NR | NR | NR | NR | 1. Married/de facto: 86 (23) | 1. Employed: 131 (35) 2. Home duties: 14 (4) 3. Retired/pensioner: 34 (9) 4. Student: 4 (1) 5. Unemployed: 151 (41) 6. Unknown: 37 (10) |  |  | "The study concludes that methamphetamine significantly contributes to fatalities in Australia over a five-year period. Cardiovascular and cerebrovascular pathologies are common among decedents. Users should be informed about the cardiotoxicity of methamphetamine and risks of polydrug use. The findings highlight the need for awareness regarding the dangers of methamphetamine use." |
| Kaye 2009 | Retrospective cohort | Australia | MDMA related death | 82 | 1. MDMA: 82 (100) | 1. Methamphetamine/amphetamine 2. Morphine 3. Alcohol 4. Codeine 5. Benzodiazepines 6. Antidepressants 7. THC 8. Cocaine/benzoylecgonine 9. Methadone 10. GHB 11. Ketamine | 14 (17) | 26 | 8.17 | NR | NR | NR | NR | NR | NR | NR | NR | 1. Employed: 57 (70)  2. Retired/pensioner: 2 (2)  3. Student: 7 (9)  4. Unemployed: 8 (10)  5. Unknown: 8 (10) | 1. Oral: 80 (98) 2. Intravenous: 2 (2) |  | "The study highlights the significant contribution of MDMA to fatalities in Australia, with 82 cases identified over five years. Cardiovascular pathology was prevalent, detected in 58% of decedents, indicating serious health risks associated with MDMA use. The findings suggest a need for user education regarding the harms of MDMA, especially when combined with other substances. Future research should explore MDMA-related morbidity and mortality in the context of other risk factors. The study emphasizes that MDMA-related deaths do not occur exclusively in specific environments, challenging common assumptions." |
| Kittirattanapaiboon 2010 | Retrospective cohort | Thailand | Meth | 449 | 1. Meth: 449 (100) | 1. Alcohol 2. Methamphetamine 3. Inhalants 4. Cannabis 5. Opioids/heroin | 42 (9.4) | NR | NR | NR | NR | 4.3 | 3.2 | NR | NR | 1. Education (y):7.6 ±3.3 | 1. Never married: 208 (46.3) | 1. Unskilled laborers/unemployed: 248 (55.2) | 1. Smoking: 396 (88.2) 2. Oral: 33 (7.3) 3. Sniffing: 17 (3.8) 4. Injection: 3 (0.7) 5. Ever injected (lifetime): 5 (1.1) |  | "The study concludes that patients with methamphetamine psychosis (MAP) face poor long-term outcomes, including premature death and chronic psychosis. Individuals with MAP require long-term psychiatric care and monitoring similar to other chronic diseases. Education is essential for MAP users to understand the need to cease drug use and recognize psychotic symptoms. The findings highlight a high rate of premature death, with suicide being a leading cause among younger participants. The study emphasizes the need for identifying high-risk groups to prevent chronicity in MAP. Participants showed significant rates of alcohol use disorder and suicidality, necessitating ongoing support." |
| Kuo 2011 | Retrospective cohort | Taiwan | Meth | 1254 | 1. Meth: 1254 (100) | NR | 245 (19.5) | 28.4 | 7.6 | NR | NR | NR | NR | NR | NR | 1. College and above: 76 (6.1) 2. Senior high: 608 (48.5) 3. Junior high and below: 570 (45.4) | 1. Single: 806 (64.3) 2. Married: 224 (17.9) 3. Others: 224 (17.9) | 1. Lifetime Employed: 1,111 (88.6) |  |  | "Patients with methamphetamine dependence have a sixfold increased risk of mortality compared to the general population. Unnatural deaths are particularly concerning in the short-term post-discharge period. A significant proportion of deceased patients died from natural causes, indicating a need for clinical attention. Women exhibit a higher standardized mortality ratio for unnatural deaths than men, highlighting a larger health gap. The study provides valuable insights for developing prevention and intervention programs for methamphetamine users." |
| Lee 2021 | Case control | Taiwan | Meth | 346 | 1. Meth: 346 (100) | NR | 60 (17.3) | 31.3 | 7.5 | NR | NR | NR | NR | NR | NR | 1. Higher than high school: 94 (27.2) | 1. Married: 61 (17.6)  2. Single: 213 (61.6)  3. Divorced/Separated: 72 (20.8) |  |  |  | "The study demonstrates that METH use partially mediates the association between ACEs and attempted suicide. Addressing METH use in individuals with ACEs could potentially reduce their suicide risk." |
| Marshall 2011 | Prospective Cohort | Canada | Meth Attempted suicide | 149 | 1. Meth: 1873 (100) | NR | 72(48.3) | 35.7026 | 10.4804 | NR | NR | NR | NR | NR | NR | NR | NR | NR | 1. Injection: 1873 (100) | 1. Caucasian: 73(49.0) 2. Aboriginalc: 65(43.6)  3. Asian: 7 (4.7)  4. Other: 4(2.7) | "The study concludes that MA injection significantly increases the risk of suicide attempts among injection drug users. It suggests that IDUs who inject MA should be closely monitored for suicidal behavior. Improved integration of mental health and suicide prevention interventions within drug treatment programs is recommended. The findings indicate that the observed incidence of attempted suicide may be underestimated due to stigma. Limitations include the inability to control for several previously identified risk factors for suicide." |
|  |  |  | Meth Did not attempt suicide | 1724 |  | NR | 605(35.1) | 39.6499 | 9.6451 | NR | NR | NR | NR | NR | NR | NR | NR | NR |  | 1. Caucasian: 1042(60.8)  2. Aboriginalc: 536(31.3) 3. Asian: 59 (3.4) 4. Other: 78(4.5) |  |
| McKetin 2021 | Prospective Cohort | Australia | Crytal Meth | 151 | 1. Crystal Meth: 151 (100) | 1. Benzodiazepines 2. Antidepressants 3. Antipsychotics | 60 (40) | med 38 | NR | med 20 | NR | 14.7026 | 10.479 | NR | NR | 1. Years of schooling: median 11y 2. Tertiary qualifications -Nil: 43 (29) -University: 16 (11) -Trade/technical: 90 (60) | 1. Single: 57 (38) | 1. Unemployed: 84 (56) |  |  | "In Australia, people who smoke crystalline methamphetamine tend to be younger and less marginalized than those who inject the drug. Despite these demographic differences, both groups exhibit similarly severe clinical profiles. This suggests that treatment and harm reduction strategies should adapt to accommodate this broader client base. The rise in methamphetamine smoking may reflect a birth cohort effect influencing new substance users. However, the potential transition from smoking to injecting remains a concern, warranting further research." |
| Paknahad 2021 | Retrospective cohort | Iran | Meth related death | 1389 | 1. Meth: 1389 (100) | 1. Methadone 2. Tramadol 3. Morphine 4. Tricyclic antidepressants 5. Benzodiazepines | NR | 37.47 | 10.87 | NR | NR | NR | NR | NR | NR | NR | NR | NR | NR | NR | "The study found that methamphetamine-related deaths in Tehran, Iran, surged tenfold between 2011 and 2018. Multidrug toxicity was the leading cause, with accidental overdoses accounting for most fatalities. Notably, younger individuals—especially males—were disproportionately affected. These findings highlight the growing public health crisis linked to methamphetamine use and its rising mortality burden in Iran." |
| Zarrabi 2016 | Cross-Sectional | Iran | Meth induced psychosis | 152 | 1. Meth: 152 (100) | NR | 13 (8.6) | 36.7 | 8.6 | NR | NR | NR | NR | NR | NR | 1. Illiterate: 12 (7.8)  2. <High school: 119 (78.3)  3. High school: 20 (13.2)  4. University graduate: 1 (0.7) | 1. Married: 49 (32.2)  2. Single: 103 (67.8) |  |  |  | "Violence in patients with methamphetamine-induced psychosis (MIP) is both severe and highly frequent. Recovery from psychotic symptoms takes longer in Iran, mirroring global trends. Preliminary research suggests electroconvulsive therapy may be effective for treating persistent psychosis and reducing violent behaviors. The study emphasizes the damaging effects of methamphetamine abuse on mental health and its contribution to violent crime in Iran. These findings should serve as an urgent warning for psychiatrists and judicial authorities to implement preventive measures." |
| Ali 2024 | Cross-Sectional | Saudi Arabia | Meth | 26 | 1. Meth: 26 (100) | NR | 5 (19.2) | 27.6 | 5.4 | NR | NR | NR | NR | NR | NR | NR | NR | NR | NR | NR | "The study concluded that impulsivity and suicidal risk were significantly higher among patients with methamphetamine use disorders, regardless of whether they used other substances. It was noted that the risk of aggression in these patients was not greater than that of individuals abusing other substances or those with polysubstance use disorders. The findings suggest that methamphetamine use disorder may represent a distinct category of substance dependence, highlighting the need for targeted interventions. Limitations included a small sample size and potential biases due to the treated community from which participants were drawn." |
|  |  |  | Polysub. with meth | 68 |  | NR | 30 (44.1) | 29 | 6.1 | NR | NR | NR | NR | NR | NR | NR | NR | NR | NR | NR |  |
| Al-Imam 2023 | Cross-Sectional | Iraq | Crystal meth | 165 | 1. Meth: 165 (100) | 1. Crystal meth alone: (55.2%) 2. Combination with alcohol (24.8%), other drugs (20%) | 13 (7.9) | 26.62 | 0.53 | NR | NR | >1 | NR | NR | NR | 1. Primary/intermediate education: 131 (79.4) 2. Secondary/college education: 25 (15.2) 3. Illiterate: 9 (5.5) | 1. Single: 85 (51.5) 2. Married: 80 (48.5) | 1. Blue-collar occupations: 134 (81.2) 2. Military/security forces: 17 (10.3) 3. Unemployed/students: 14 (8.5) | 1. Snorting only: 92 (55.8) 2. Oral + snorting: 71 (43.0) 3. Oral + smoking: 2 (1.2) |  | "The study identified several key risk factors associated with suicidal ideation among Iraqi crystal methamphetamine users, including prolonged use exceeding one year and the presence of poly-drug abuse. It emphasized the importance of an interdisciplinary approach involving mental health professionals to monitor and evaluate at-risk individuals. The findings highlighted the critical societal issue of substance abuse in Iraq, affecting the quality of life for many individuals and their families. The study underscored the need for tailored interventions to mitigate the impact of crystal methamphetamine use on mental health." |
| Artenie 2014 | Cross-Sectional | Canada | Polysub. (Attempted suicide) | 71 | 1. AMPH a.Chronic: 26(2.3)  b.Occasional: 12(17.4) | 1. Cocaine 2. Amphetamine 3. Opioids 4. Sedative-hypnotics 5. Cannabis 6. Alcohol | 19(26.8) | 39.1233 | 15.2856 | 21.7637 | 8.3239 | 13.1459 | 9.2319 | NR | NR | 1. Completed high-school education a.Yes: 42(59.2)  b.No: 29(40.8) |  |  |  |  | "The study indicates a unique link between stimulant use and suicide attempts among persons who inject drugs (PWID). Stimulant-using PWID should be a primary focus for suicide prevention efforts. Future research should explore the neurobiological and social factors influencing suicide risk in this group. The role of overlapping substance use in relation to suicidal behaviors warrants further investigation. Findings suggest that stimulant use significantly elevates the odds of suicide attempts compared to opioids. The study highlights the need for targeted interventions for stimulant users within the PWID population." |
|  |  |  | Polysub. (did not Attempted suicide) | 1169 | 1. AMPH: a.Chronic: 26(2.3) b.Occasional: 151(13.1) | 1. Cocaine 2. Amphetamine 3. Opioids 4. Sedative-hypnotics 5. Cannabis 6. Alcohol | 183(15.7) | 38.2946 | 11.3561 | 22.101 | 8.9067 | 13.6104 | 10.8365 | NR | NR | 1. Completed high-school education a.Yes: 704(60.2)  b.No: 465(39.8) |  |  |  |  |  |
| Baberg 1996 | Cross-Sectional | USA | AMPH Users | 259 | 1. AMPH: 259 (100) | NR | 82 (31.8) | NR | NR | NR | NR | NR | NR | NR | NR | NR | 1. Single: 164 (67.2)  2. Divorced, separated: 49 (20.1)  3. Married, widowed: 31 (12.7) |  |  | 1. White: 180 (71.3) 2. Asian: 1 (0.4)  3. Black: 35 (13.5)  4. Hispanic: 36 (14.3) | "In summary, amphetamine use is found in a large and growing number of patients seen on a San Diego psychiatric consultation service. The demographic data indicate that young white men are especially likely to be amphetamine users. However, there were users in all age groups (the youngest was 14 years of age, and the oldest 70), in nearly all wards, and with a variety of symptoms. The high prevalence of amphetamine use could emerge unexpectedly in other regions of the country, and thus the possibility of undiagnosed amphetamine use must be considered with regard to all patients seen in psychiatric consultation." |
|  |  |  | Non-users | 2724 |  | NR | 1231 (45.5) | NR | NR | NR | NR | NR | NR | NR | NR | NR | 1.Single: 1,278 (48.6)  2. Divorced, separated: 574 (21.8)  3. Married, widowed: 779 (29.6) |  |  | 1. White: 1,739 (64.9)  2. Asian: 90 (3.4)  3. Black: 355 (13.3)  4. Hispanic: 493 (18.4) |  |
| Brecht 2004 | Cross-Sectional | USA | Meth | 350 | 1. Meth: 350 (100) 2. Ecstasy: 70 (20) | 1. Alcohol 2. Marijuana 3. Tobacco 4. Cocaine 5. Hallucinogens 6. Crack 7. Inhalants 8. PCP 9. Heroin 10. Tranquilizers 11. Downers 12. Other opiates 13. Ecstasy | 154 (44) | NR | NR | 18.98 | 5.58 | 2.14 | 4.15 | NR | NR | 1. Less than high school diploma: 112 (32.00) 2. At least some college: 112 (32.00) 3. High school: 74 (21.00) 4. Trade/tech school: 53 (15.00) |  |  | 1. Snort: 154 (44.00) 2. Smoke: 119 (34.00) 3. Inject: 70 (20.00) 4. Eat/drink: 7 (2.00) | 1. Non-Hispanic White: 161 (46.00) 2. Hispanic: 102 (29.00) 3. African American: 56 (16.00) 4. Other: 28 (8.00) | "The paper concludes that methamphetamine (MA) use behaviors exhibit both similarities and significant gender differences, which have implications for treatment and prevention strategies. It highlights that females are more likely to be introduced to MA through intimate relationships, while males often initiate use through friends. The study emphasizes the need for gender-specific approaches in treatment, as females report different MA-related problems compared to males. Additionally, the findings suggest a concerning trend of early substance use initiation and rapid escalation to regular use, necessitating ongoing research to monitor these dynamics over time." |
| Chen 2007 | Cross-Sectional | Taiwan | Meth Suicide attempts (+) | 108 | 1. Meth: 439 (100) | 1. Opiates 2. Cocaine 3. Marijuana 4. Organic solvents 5. Sedatives | 62 (38.3) | 27.5 | 6.7 | 21.1 | 6.7 | 2.883 | 2.53 | NR | NR | 1. Education (y): 9.3 ± 2.1 |  | NR | 1. Inhalation: 437 (99.54) 2. Injection: 2 (0.46) | 1. Chineese: 439 (100) | "The study concluded that among Chinese methamphetamine (MAP) abusers, suicide attempts were significantly associated with female gender, history of MAP-induced psychotic and depressive disorders, and family history of psychotic disorders. The triallelic 5-HTTLPR polymorphism was not found to be associated with suicidal behavior, MAP-induced depressive disorders, or MAP-induced psychotic disorders in the sample studied. The findings suggest that the association of the 5-HTTLPR polymorphism with suicidal behavior may not exist among MAP abusers, particularly in the Taiwanese population. The study highlights the need for further research with larger sample sizes to explore these associations." |
|  |  |  | Meth Suicide attempts (–) | 331 |  |  | 100 (61.7) | 27.2 | 6.1 | 21.8 | 7 | 2.45 | 2.275 | NR | NR | 1. Education (y): 9.3 ± 2.4 |  | NR |  |  |  |
| Cloutier 2013 | Retrospective cohort | USA | Meth | 130 | 1. Meth: 130 (100) | NR | 48 (36.9) | 34.4 | 10.7 | NR | NR |  |  | NR | NR | NR | NR | NR | NR | 1. White:108 (83.1)  2. Non-white: 22 (16.9) | "Patients with methamphetamine-related psychiatric visits were younger and less likely to have a history of major depressive disorder. The groups showed no significant differences in demographic, historical, or vital sign information. Methamphetamine-related visits accounted for 7.6% of all psychiatric ED visits. The study highlights the need for better understanding of methamphetamine's impact on psychiatric emergencies." |
| Demir 2021 | Retrospective cohort | Turkey | Meth/AMPH | 90 | 1. Meth/AMPH: 90 (100) | NR | NR | 26.1 | 6 | 19.9 | 4.7 | 7.8 | 7.7 | NR | NR | 1. Illiterate: 8 (8.9) 2. Primary school: 67 (74.4) 3. High school: 10 (11.1) 4. University: 3 (3.3) 5. Non-illitrate: 2 (2.2) | 1. Single: 56 (65.7)  2. Married: 32 (35.6) 3. Divorced: 2 (2.2) | 1. Do not work: 29 (32.2) 2. Irregular work: 58 (64.4) 3. Regular work: 3 (3.3) |  |  | "The study is the first conducted in the Gaziantep AMATEM Clinic, the only inpatient drug and alcohol center in Southeastern Anatolia. Alcohol and substance use negatively impacts work, education, family, and social relations, necessitating effective policy solutions. Understanding addiction and treatment factors is crucial for developing effective policies and resource utilization. The findings highlight the dynamic nature of substance use problems and their broad societal effects. The study aims to inform treatment and rehabilitation policies based on the characteristics of substance use in the region." |
| Derlet 1989 | Retrospective cohort | USA | Meth | 127 | 1. Meth: 127 (100) + additional sub. NB. Only meth: 65 (53.7) | 1. Alcohol 2. Benzodiazepines 3. Aspirin 4. Cocaine 5. Ephedrine 6. Phenytoin 7. Phenylpropanolamine 8. Diphenhydramine | 45 (35) | 29.9 | 5.7 | NR | NR |  |  | NR | NR | NR | NR | NR | 1. IV: 44 (35),  2. Oral ingestion: 18 (14) 3. Nasal “snorting”: 12 (10) 4. Unknown: 52 (41) |  | "The study concludes that the majority of patients presenting with amphetamine toxicity displayed significant alterations in mental status, primarily agitation and confusion. Most cases were managed conservatively in the emergency department, with only 10% requiring pharmacologic intervention. Admission to the hospital was necessary for a small number of patients, primarily to rule out serious complications such as myocardial infarction or stroke. The findings highlight the variability in response to amphetamines and the challenges in determining the exact dose necessary to produce toxic effects. Overall, clinical observation of symptoms is deemed more relevant than estimating the ingested dose." |
| Fass 2009 | Cross-Sectional | USA | Meth | 28 | 1. Meth: 28 (100) | 1. Cigarette  2. Alcohol | 13 (46.4) | 15.6 | NR | NR | NR | NR | NR | NR | NR | NR | NR | NR | 1. Smoked meth: 24 (85.7) 2. Injected meth: 2 (7.1) 3. Snorted meth: 2 (7.1) | 1. Caucasian: 25 (89.3) 2. Latino: 1 (3.6) 3. Other: 2 (7.1) | "The study concludes that detained adolescent methamphetamine users demonstrate significantly different characteristics and risk behaviors compared to other drug-using youth, exhibiting higher rates of substance abuse, delinquency, impulsivity, anxiety, depression, and suicidal tendencies. These findings highlight the critical need for tailored treatment interventions that address the distinct psychological profile of adolescent meth users. However, researchers caution that these results must be interpreted within the study's limitations, including its small sample size and specific focus on a detained population, which may affect generalizability to broader adolescent groups." |
|  |  |  | Other Drugs | 25 |  |  | 7 (28) | 15.5 | NR | NR | NR | NR | NR | NR | NR | NR | NR | NR |  | 1. African American: 10 (40.0) 2. Latino/a: 1 (4.0) 3. Caucasian: 13 (52.0) 4. Other: 1 (4.0) |  |
| Frankeberger 2024 | Prospective Cohort | USA | Current only Meth Use | 20 | 1. Meth: 20 (100) | 1. Marijuana 2. Cocaine or Crack 3. Sedatives | 20 (100) | 33.4 | 3 | NR | NR | NR | NR | NR | NR | 1. 8th Grade or Less: 1 (5)  2. Less than High School Degree: 9 (45)  3. High School Degree or GED: 9 (45)  4. Some College or More: 1 (5) | 1. Single, never married: 9 (45)  2. Married or Living Together: 6 (30)  3. Separated, Divorced, or Widowed: 5 (25) | 1. Current Employment: 11 (55) |  |  | "The study highlights how the overdose crisis disproportionately impacts racial and ethnic minoritized people who use drugs, particularly low-income Latina women, underscoring the need for prevention strategies that adopt holistic approaches addressing socioeconomic and mental health contexts. Findings reveal that social, structural, and mental health inequities significantly exacerbate drug use risks, emphasizing the urgent requirement for culturally responsive treatment access for marginalized populations. Notably, women using methamphetamine show significantly higher likelihood of reporting suicidal ideation, calling attention to the critical need to address co-occurring mental health conditions among substance users. The research ultimately advocates for comprehensive, multi-faceted strategies to effectively combat polysubstance use and its associated harms across vulnerable communities." |
| Hadinezhad 2019 | Cross-Sectional | Iran | Meth | 39 | 1. Meth: 39 (100) | NR | 7 (18) | 31.23 | NR | NR | NR | NR | NR | NR | NR | 1. Below High School Diploma: 14 (35.9) 2. High School Diploma: 14 (35.9) 3. Undergraduate/Bachelor's Degree: 11 (28.2) | 1. Married: 15 (38.5) 2. Single: 20 (51.3) 3. Divorced: 4 (10.3) |  |  |  | "Methamphetamine use is particularly prevalent among young men in their second and third decades of life, with common presenting symptoms including hypertension, chest pain, palpitations, and seizures. The research underscores both the significant psychiatric and medical complications associated with methamphetamine use and the pressing need for improved identification of users in emergency department settings to ensure appropriate care and intervention." |
| Hypse 2018 | Cross-Sectional | USA | Meth | 28 |  | NR | 9 (32) | 33 | 9.97 | 19.2 | 6.68 | NR | NR | NR | NR | 1. 8th Grade: 2 (7.1) 2. 9th Grade: 2 (7.1) 3. 10th Grade: 2 (7.1) 4. 11th Grade: 2 (7.1) 5. 12th Grade/High School Graduate: 4 (14.3) 6. Some College: 11 (39.3) 7. Associates Degree: 1 (3.6) 8. Bachelor’s Degree: 3 (10.7) 9. Graduate School: 1 (3.6) |  | 1. No Work History: 5 (17.9) 2. Mental Health: 2 (7.1) 3. Substance Abuse: 3 (10.7) 5. Business: 2 (7.1) 6. Service Industry: 7 (25) 7. General Labor: 9 (32.1) | 1. IV: 18 (64) |  | "Frequent methamphetamine use, particularly intravenous administration, shows a significant correlation with higher rates of suicide attempts among users. However, this relationship appears complex and non-traditional, as many participants reported suicide attempts predating their first meth injection. The study suggests that unidentified underlying factors—beyond direct drug effects—may contribute to the link between IV meth use and suicidal ideation. These findings underscore the critical need for deeper investigation into the psychosocial, biological, and behavioral variables that connect methamphetamine use patterns with suicide risk. Notably, the research consistently identifies a strong association between intravenous meth use and suicide attempts, warranting urgent clinical attention." |
| Kalayasiri 2009 | Cross-Sectional | USA | Meth induced Paranoia | 44 | 1. Meth: 96 (100) | 1. Tobacco dependence 2. Alcohol dependence 3. Marijuana 4. Solvents 5. Combination drugs 6. Opiates 7. Hallucinogens 8. Sedatives 9. Stimulants | 28 (63.6) | 25.2 | 4.3 | 17.6 | 4.1 | 1. 1 - 3y: 23 (52.3)  2. ≥ 4y: 21 (47.7) |  | 3.2y | 3.3 | 1. Grade 0-8: 25 (56.8)  2. Grade ≥ 9: 19 (43.2) | 1. Married or living-together: 11 (25.0)  2. Widowed / separated/divorced: 23 (52.3)  3. Never married or living together: 10 (22.7) | 1. Employed: 7 (15.9) | 1. Inhalation/smoking: 96 (100)  2. Oral: 12 (12.5) 3. Injection: 0 (0) | NR | "Severity of dependence/use was an important risk factor with respect to the occurrence of MIP and its prolonged symptomatic expression. Conversely, the phenotypic expression of MIP at earlier stages may be a moderating factor with respect to overall MA consumption. That being said, future studies (including prospective, and longitudinal designs) will be required to clarify more precisely the nature of such associations. Similarly, future studies of genetic factors will be important in conjunction with such efforts, enabling a more comprehensive understanding of potential, and most probably important, gene by environment interactions that are likely to underlie the phenotypic expression of the trait." |
|  |  |  | Non MIP | 52 |  |  | 35 (67.3) | 26.4 | 5.2 | 19.9 | 7.1 | 1. 1 - 3y: 35 (67.3)  2. ≥ 4y: 17 (32.7) |  |  |  | 1. Grade 0-8: 31 (59.6)  2. Grade ≥ 9: 21 (40.4) | 1. Married or living-together: 22 (42.3)  2. Widowed / separated / divorced: 20 (38.5) 3. Never married or living-together: 10 (19.2) | 1. Employed: 3 (5.8) |  | NR |  |
| Kalechstein 2000 | Cross-Sectional | USA | Polysub. | 1580 | 1. Meth: 170 (10.7) | 1. Methamphetamine 2. Powder cocaine 3. Crack cocaine 4. Marijuana 5. Heroin 6. Alcohol | 378 (23.9) | 31.4 | 8.6 | NR | NR | NR | NR | NR | NR | 1. 12 years or fewer: 1,132 (71.6) 2. 13 years or more: 445 (28.4) |  |  |  | 1. Black: 398 (25.2) 2. White: 540 (34.2) 3. Latino: 534 (33.8) 4. Other: 114 (7.2) | "The study found that methamphetamine-dependent individuals, especially women, face significantly higher risks of depression and suicidal ideation compared to non-dependent populations. These associations persisted even after accounting for demographic variables and co-occurring substance use disorders. The research underscores critical gaps in our understanding of psychiatric symptoms linked to methamphetamine dependence and calls for more comprehensive investigation of these mental health impacts. Importantly, the findings highlight an urgent need to integrate specialized psychiatric care into treatment programs for methamphetamine users to address their complex psychological needs effectively." |
| Karabulut 2023 | Cross-Sectional | Turkey | Meth | 95 | 1. Meth: 95 (100) | 1. Tobbaco | 19 (20) | 29.4 | 6.6 | 26.4 | 6.5 | 2 | 1.61 |  |  | 1. Secondary school (8 years): 49 (51.6) 2. High school (12 years): 25 (26.3) 3. University: 4 (4.2) | 1. Single: 43 (45.3)  2. Married/In a relationship: 33 (34.7) 3. Divorced: 19 (20) | 1. Unemployed: 44 (46.3)  2. Temporary employee: 10 (10.5)  3. Regular employee: 38 (40)  4. Works with family: 3 (3.2) | 1. Smoking: 87 (91.6) 2. Injection: 4 (4.2) 3. Oral: 2 (2.1) 4. Intranasal: 2 (2.1) | NR | "The study concludes that assessing depressive and psychotic symptoms in methamphetamine users is crucial for effective treatment strategies. It identifies suicide as a significant predictor of both depressive and psychotic symptoms, emphasizing the need for thorough suicide evaluations upon admission. The amount of methamphetamine used is also highlighted as a predictor of these symptoms, with chronic binge smokers at higher risk for substance-related depression and psychosis. The research contributes valuable insights into methamphetamine use characteristics in Turkey, indicating a need for further prospective studies." |
| Li 2023 | Cross-Sectional | China | Meth Non-depressed group | 239 | 1. Meth: 613 (100) | NR | 0 (0) | 34.3 | 7 | 25.2 | 7.9 | NR | NR | NR | NR | 1. Education (yrs): 9±2 | 1. Single: 96 (40)  2. Married: 70 (29.2) 3. Divorced: 73 (30.5) 4. Widowed: 0 (0) |  |  | NR | "The study concludes that male patients with methamphetamine use disorder (MAUD) exhibit a high incidence of depressive symptoms, which are associated with greater drug cravings and aggression. It highlights that depressive symptoms may play a significant role in the relationship between drug craving and aggression in these patients. The findings suggest that interventions addressing depressive symptoms could be beneficial in alleviating drug cravings and managing aggression, indicating a need for psychological care beyond just drug abstinence. The study emphasizes the importance of further research to validate these findings and explore the effects of depression on drug-related behaviors." |
|  |  |  | Meth depressed group | 374 |  | NR | 0 (0) | 34.4 | 6 | 25.1 | 7.1 | NR | NR | NR | NR | 1. Education (yrs): 9±2 | 1. Single: 151 (40.37) 2. Married: 107 (28.6) 3. Divorced: 114 (30.48) 4. Widowed: 1 (0.26) |  |  | NR |  |
| Lin 2004 | Cross-Sectional | Taiwan | Meth | 325 | 1. Meth: 325 (100) | 1. Sedatives  2. Opiates  3. Marijuana  4. Solvents 5. Cocaine | 145 (44.6) | 26.8 | 7 | 21.6292 | 6.8888 | 2.2 | 2.08 | NR | NR | NR | 1. Married: 57 (17.5) 2. Never married: 214 (65.8) 3. Divorced/separated/widowed: 66 (16.6) | 1. Employed: 282 (87.9) 2. Unemployed: 94 (12.1) |  | 1. Chineese: 325 (100) | "The study found that 22.1% of methamphetamine (MAP) abuse subjects met criteria for past MAP-induced psychotic disorder. Most subjects experienced psychotic symptoms that subsided within one month after stopping MAP use. Prolonged MAP psychosis exists but may be over-emphasized in hospital-based studies. Women reported more mental disturbances and treatment-seeking behaviors compared to men. The study highlights the need for targeted prevention efforts for individuals with prior psychiatric disorders. Gender differences in psychiatric disorders and treatment-seeking behaviors were noted among MAP abusers." |
| Massah 2019 | Cross-Sectional | Iran | Crysal Meth | 82 | 1. Meth: 82 (100) | NR | 82 (100) | 31 | 7 | NR | NR | NR | NR | NR | NR | NR | NR | NR | NR | NR | "The study concludes that suicide attempts are significantly higher in females using crystal methamphetamine compared to opium users. It emphasizes the need for preventive plans and emergency interventions for at-risk populations. The research highlights limitations due to its cross-sectional design and lack of comparable studies. Further research with improved methodologies is recommended to validate these findings." |
| Miller 2016 | Cross-Sectional | USA | Polysub. | 2333 | 1. Meth: 103 (4.4) | 1. Ethanol  2. THC  3. Cocaine 4. Meth.  5. Mult. Sub.  6. Benz.  7. Opiate  8. Barb. 9. PCP | 1305 (55.9) | 36.9 | 12.8 | NR | NR | NR | NR | NR | NR | NR | NR | NR | NR | 1. Caucasian: 2065 (88.5) | "The study supports the clinical observation that patients actively using substances may stabilize quickly regarding suicidal urges and their need for inpatient care. It suggests that risk mitigation for these patients may be best achieved through substance use disorder (SUD) treatment. The findings indicate that patients with SUDs who are not currently intoxicated may be at a higher risk of suicide, warranting further research. Overall, the study emphasizes the importance of understanding the relationship between active substance use and suicide risk in psychiatric inpatients." |
| McKetin 2011 | Cross-Sectional | Australia | Meth | 400 | 1. Meth: 400 (100) | 1. Tobacco  2. Alcohol  3. Cannabis 4. Ecstasy  5. Cocaine  6. Heroin  7. Inhalants | 104 (26) | 31.1712 | 6.429 | med 17y |  | med 11 | NR | NR | NR | NR | 1. Married: 68 (17) | 1. Unemployed: 324 (81) | 1. Injection: 340 (85) | NR | "The study revealed alarmingly high rates of depression among individuals entering methamphetamine treatment, underscoring a critical clinical issue. Specifically, 40% of participants met DSM-IV criteria for a major depressive episode in the past year. An additional 44% exhibited severe substance-induced depressive symptoms. These findings highlight an urgent need for standardized clinical protocols to address depression in methamphetamine treatment programs, ensuring comprehensive mental health support alongside addiction care." |
| Nazari 2023 | Cross-Sectional | Iran | Meth psychotic | 93 | 93 (100) | 1. Opium 2. Heroin  3. Cannabis  4. Cigarette  5. Alcohol | 26 (48.1) | 36.54 | 8.2 | 21.3 | 8.2 | 14.03 | 8.16 | NR | NR | 1. Illiterate: 27 (13.6) 2. Primary education: 48 (24.2) 3. Middle school: 68 (34.3) 4. Secondary education: 12 (6.1) 5. High school diploma: 29 (14.6) 6. Associate degree and higher: 14 (7.1) | 1. Married: 95 (48) 2. Single: 50 (25.3) 3. Divorced: 40 (20.2) 4. Widow/Widower: 6 (3) 5. Second marriage (with a history of previous divorce): 7 (3.5) | 1. Employed: 117 (59.1) 2. Unemployed: 78 (38.9) | 1. Smoking: 171 (73.2) 2. Oral: 14 (7.1) 3. Injection: 13 (4.5) | NR | "Methamphetamine users often experience delusions and high-risk behaviors, with auditory and visual hallucinations being particularly common. Significant differences exist between psychotic and non-psychotic methamphetamine users in terms of duration of use and psychiatric history. A thorough evaluation of both substance use history and psychotic symptoms is crucial for developing targeted therapy plans. Secondary preventive measures are essential to reduce harm and prevent psychosis from methamphetamine use. Early detection of psychosis symptoms can help prevent severe individual and social complications." |
|  |  |  | Meth non-psychotic | 105 | 105 (100) |  | 28 (51.9) | 36.28 | 8.61 | 36.28 | 8.65 | 10.56 | 28.8 | NR | NR |  |  |  |  | NR |  |
| Njuguna 2021 | Retrospective cohort | USA | Meth/AMPH-involved overdose | 1066 |  | 1. Alcohol 2. Cannabis 3. Cocaine 4. Opioid 5. Sedative 6. Tobacco 7. Heroin | 384 (36) | 40.8 | 10 | NR | NR | NR | NR | NR | NR | NR | NR | NR | NR | NR | "The study found increasing rates of METH/AMPH-involved overdose hospitalizations in Washington State, particularly among older adults. Polysubstance use, including methamphetamine, contributes significantly to overdose hospitalizations. Interventions tailored for older persons could effectively reduce METH/AMPH-involved overdose hospitalizations. The age-adjusted hospitalization rate for METH/AMPH increased from 6.3 to 8.5 per 100,000 persons from 2010 to 2017. A significant proportion of hospitalized patients had substance use disorders, indicating a need for comprehensive treatment approaches." |
| Roxburgh 2020 | Retrospective cohort | Australia | MDMA related death | 392 | 1. MDMA: 392 (100) | NR | 74 (19) | 36.8 | 7.2903 | NR | NR | NR | NR | NR | NR | NR | 1. Married/Defacto: 77 (20) | 1. Employed: 242 (62) |  | NR | "MDMA-related deaths predominantly occurred among males in their mid-twenties, with females being significantly younger than males. Most incidents of death occurred in private locations, with only a small number at music festivals or dance parties. There were three distinct periods of increases and declines in death rates, aligning with international MDMA supply trends. A significant proportion of deaths were due to multiple drug toxicity, while a notable number were attributed solely to MDMA toxicity.  Engagement with young consumers regarding the risks of MDMA use is critical, especially in light of increasing drug purity." |
| Park 2022 | Cross-Sectional | USA | Meth | 219 | 1. Meth: 219 (100) | 1. Psychostimulants 2. Methamphetamine 3. Cocaine 4. PMA 5. Other 6. Alcohol 7. Opioids 8. Cannabis 9. Benzodiazepines 10. Antidepressants 11. Ketamine 12. GHB 13. Antipsychotics | 95 (43.4) | 37.8 | 12 | NR | NR | NR | NR | NR | NR | 1. High school: 89 (40.63) 2. Some college: 69 (31.51) 3. Less than high school: 51 (23.29) 4. College or higher: 10 (4.57) | 1. Never married: 110 (50.23) 2. D/S/W: 71 (32.42) 3. Married: 38 (17.35) | 1. Employed: 98 (44.75) 2. Not in labor force: 75 (34.25) 3. Unemployed: 46 (21.00) | 1. Injection: 219 (100) | 1. White: 158 (72.15) 2. Other: 37 (16.89) 3. Hispanic: 17 (7.76) 4. Black: 7 (3.20) | "The study identifies three distinct groups of people who inject drugs (PWID) with unique drug use patterns and behavioral health comorbidities. A majority of PWID (80.2%-85.9%) had an illicit drug use disorder, yet only one-fourth received treatment. The methamphetamine injection group faces higher risks of psychological distress and limited access to treatment. Targeted interventions are necessary to improve treatment access for PWID, especially those experiencing homelessness. The findings highlight the need for expanded harm reduction programs tailored to the socioeconomic profiles of at-risk communities." |
| Peng 2025 | Cross-Sectional | China | Meth without Gambling disorder | 340 | 340 (100) | 1. Cannabis  2. Simulant  3. Sedative  4. Ketamine  5. Alcohol 6. Opioid 7. Tobacco  8. Nicotine | 48 (14) | 33.7011 | 7.4449 | 26.0517 | 8.1894 | NR | NR | NR | NR | NR | 1. Married: 119 (35)  2. Single or divorced: 220 (65) | 1. Employed: 145 (43) |  | NR | "The study concludes that gambling disorder (GD) is prevalent among individuals undergoing treatment for methamphetamine use disorder (MUD) in China, with 52.3% of MUD participants meeting the DSM-5 criteria for GD. Significant correlates of GD include concurrent alcohol use, childhood violent experiences, major depressive episodes (MDEs), severe MUD, and gambling duration. Network analysis identifies gambling preoccupation and methamphetamine tolerance as central features, with methamphetamine tolerance acting as a bridge between GD and MUD. These findings highlight the need for targeted interventions to address the co-occurrence of GD and MUD." |
|  |  |  | Meth + GD | 371 | 371 (100) |  | 28 (7.5) | 31.7011 | 5.9538 | 24.3505 | 6.698 | NR | NR | NR | NR | NR | 1. Married: 144 (39) 2. Single or divorced: 227 (61) | 1. Employed: 175 (47) |  | NR |  |
| Perello 2022 | Retrospective cohort | Spain | AMPH and derivatives intoxication | 170 | 1. Methamphetamine: 117 (68.9) 2. Amphetamine: 31 (18.2) 3. MMDA: 22 (12.9) | 1. Polysubstance | 0 (0) | 36.2 | 7.5 |  |  | NR | NR | NR | NR | NR | NR | NR | 1. Injection: 12 (7.1) | NR | "Amphetamine-related intoxication significantly increases morbidity in individuals living with HIV. Healthcare providers should routinely assess recreational drug use among these patients. Incorporating harm-reduction measures is essential in preventing amphetamine-related intoxications. The study highlights the need for integrated healthcare approaches addressing mental health and substance use. The findings emphasize the importance of understanding clinical characteristics of amphetamine-related intoxication in this population" |
| Richards 1999 | Retrospective cohort | USA | Meth | 461 |  | 1. Tobacco  2. Ethanol 3. Opiates  4. Cocaine  5. Benzodiazepines  6. Tetrahydrocannabinol  7. Phencyclidine | 166 (36) | 34.9 | 8.5 | NR | NR | NR | NR | NR | NR | NR | NR | NR | NR | 1. Caucasian: 341 (74)  2. Hispanic: 63 (13)  3. African American: 37 (8)  4. Asian. Pacific Islander: 18 (4) 5. Native American: 2(1) | "Methamphetamine abuse patients presenting to the ED tend to be young Caucasian males, tobacco smokers, and have no health insurance. Compared to the average ED patient, MAP patients use ambulance transport at a much higher rate and are more likely to be admitted to the hospital. A significant association between MAP patients and trauma also existed. Methamphetamine abuse will continue to worsen if current trends prevail. Clinicians must be aware of the variable nature of MAP abuse and its deleterious effect on patients' mental and physical health." |
|  |  |  | Non Meth | 32156 |  |  | 15.435 (48) | 41.2 | 13.6 | NR | NR | NR | NR | NR | NR | NR | NR | NR | NR | 1. Caucasian: 16399 (51)  2. Hispanic: 3,859 (1 2)  3. African American: 6110 (19) 4. Asian. Pacific Islander: 5,576 (1 7)  5. Native American: 212 (1) |  |
| Richards 2017 | Retrospective cohort | USA | Meth | 3013 | 1. Meth: 610 (20.24) | NR | 223 (36.6) | 41.7 | 12.4 | NR | NR | NR | NR | NR | NR | NR | NR | NR | NR | 1. Caucasian: 436 (71.4)  2. Hispanic: 102 (16.7)  3. African American: 40 (6.6) 4. Asian/Pacific Islander: 31 (5.1)  5. Native American: 1 (0.2) | "Methamphetamine use is several times more prevalent than cocaine in our ED patient population, and this prevalence is increasing compared to previous studies. Methamphetamine users were more likely to be Caucasian, present with altered levels of consciousness, be placed on 72-h psychiatric holds, or be transferred to inpatient psychiatric facilities. Cocaine users had a higher rate of trauma, alcohol intoxication, and elopement from the ED. These differences may be explained by regional preferences, socialization, personality types, and the unique neuropsychopharmacological differences between cocaine and methamphetamine" |
|  |  |  | Cocaine/Meth |  | 1. Cocaine/Meth: 28 (0.9) | NR | 6 (21.4) | 39.4 | 15.9 | NR | NR | NR | NR | NR | NR | NR | NR | NR | NR | 1. Caucasian: 15 (53.7)  2. Hispanic: 5 (17.8)  3. African American: 6 (21.4)  4. Asian/Pacific Islander: 2 (7.1) |  |
| Soboka 2024 | Cross-Sectional | Canada | Polysub. Suicide no risk | 13901 | 1. AMP/Meth: 91 (0.65) | 1. Alcohol use 2. Opioid use 3. Amphetamine/Methamphetamine use 4. Cocaine use 5. Cannabis use 6. Hallucinogen use 7. Sedatives/Hypnotics use 8. Tobacco use 9. Polysubstance use | 5453 (39.2) | 38.7 | 12.1 | NR | NR | NR | NR | NR | NR | NR | 1. Married/common-law/partnership: 7360 (52.94) 2. Single: 4486 (32.26) 3. Separated/divorced/widowed: 2055 (14.78) |  |  | 1. White: 8102 (58.28) 2. Indigenous: 525 (3.78) 3. Others: 616 (4.43) 4. Declined: 4658 (33.51) | "There was a high prevalence of suicide behaviours among individuals who were seeking MHA services. Also, this study showed that using substances such as alcohol, tobacco, stimulants, sedatives, and hallucinogens were associated with suicide risk. Health professionals need to be aware of these associations and provide comprehensive assessments and support to individuals struggling with substance use and suicide risk, addressing both underlying mental health conditions and substance use. Also, our findings highlighted the association between sedatives/hypnotics and suicide risk, emphasising the need for vigilance and comprehensive screening for individuals undergoing treatment with these medications. Our findings underscore the importance of regular screening for suicide risk among clients seeking MHA treatment services. Furthermore, our findings imply the need for targeted interventions and preventive measures for individuals engaging in substance use. Further studies are needed to understand the temporal relationship between substance use and suicide; particularly, it is essential to investigate the association between sedatives/hypnotics as well as hallucinogens and suicide risk." |
|  |  |  | Polysub. Suicide low risk | 7682 | 1. AMP/Meth: 111 (1.44) |  | 3068 (39.9) | 36.1 | 12 | NR | NR | NR | NR | NR | NR | NR | 1. Married/common-law/partnership: 3696 (48.11) 2. Single: 2922 (38.04) 3. Separated/divorced/widowed: 1064 (13.85) |  |  | 1. White: 5012 (65.24) 2. Declined: 2009 (26.15) 3. Others: 358 (4.66) 4. Indigenous: 303 (3.94) |  |
|  |  |  | Polysub. Suicide: Moderate/high risk | 917 | 1. AMP/Meth: 31 (3.4) |  | 277 (30.2) | 36.3 | 12.4 | NR | NR | NR | NR | NR | NR | NR | 1. Single: 407 (44.38) 2. Married/common-law/partnership: 365 (39.80) 3. Separated/divorced/widowed: 145 (15.81) |  |  | 1. Declined: 401 (43.62) 2. White: 445 (36.96) 3. Indigenous: 36 (3.28) 4. Others: 35 (3.47) |  |
| Stronach 2024 | Retrospective cohort | Australia | Meth-related death | 8812 | 1. Only Methamphetamine toxicity: 769 (8.7) | 1. Mixed drug toxicity includes Opioids, benzodiazepines, antidepressants | 1995 (22.63) | 37 |  | NR | NR | NR | NR | NR | NR | NR | 1. Married/de facto relationship: 2145 (24.3)  2. Never married: 3136 (35.6) | 1. Employed: 2370 (26.9)  2. Unemployed: 4111 (46.6) | 1. Injection: 3965 (45) | NR | "The study analyzes methamphetamine-related mortality in Australia from 2001 to 2020, highlighting an overall trend of escalating harm. There is an urgent need for accessible services for individuals using methamphetamine, particularly in mental health care. The increase in natural cause deaths, especially from circulatory diseases, raises long-term health concerns. A multi-layered response is necessary, integrating education, harm reduction, and healthcare support for methamphetamine users. The findings emphasize the complexities of methamphetamine-related mortality and the need for tailored strategies." |
| Toles 2006 | Retrospective cohort | Hawaii | Non-meth Diagnosis | 738 |  | 1. Alcohol 2. Cocaine 3. THC (Tetrahydrocannabinol) 4. Benzodiazepine 5. Opiate 6. Other drugs | 309 (42) | NR | NR | NR | NR | NR | NR | NR | NR | NR | NR | NR | NR | 1. Caucasian: 320 (4)  2. Filipino: 66 (9)  3. Japanese: 92 (13)  4. Native Hawaiian: 111 (15)  5. Other Asian: 72 (10) 6. Other: 62 (8) | "The study concludes that psychiatric emergency department patients with methamphetamine-related diagnoses present more acutely and utilize more hospital resources compared to those without such diagnoses. It was found that 18% of the patients had methamphetamine-related diagnoses, with a significant prevalence of dual diagnoses and co-morbid mental illnesses such as schizophrenia and major depression. Patients with methamphetamine-related issues were more likely to exhibit agitation and suicidality, leading to longer stays in the emergency department and higher rates of hospital admission. The findings highlight the urgent need for targeted interventions for this demographic." |
|  |  |  | Meth Diagnosis | 166 | 1. Meth: 166 (100) |  | 48 (29) | NR | NR | NR | NR | NR | NR | NR | NR | NR | NR | NR | NR | 1. Caucasian: 42 (25) 2. Filipino: 18 (11) 3. Japanese: 21 (13) 4. Native Hawaiian: 43 (26) 5. Other Asian: 21 (13) 6. Other: 14 (8) |  |
| Topp 1998 | Cross-Sectional | Australia | Polysub. | 329 | 1. Ecstasy: 329 (100) 2. Amphetamine: 310 (94.2) 3. MDA: 166 (50.5) | 1. Ecstasy 2. Alcohol 3. Cannabis 4. Amphetamine 5. LSD 6. Tobacco 7. Amyl nitrate 8. Cocaine 9. Nitrous oxide 10. Benzodiazepines 11. MDA 12. Other opiates 13. Heroin 14. Antidepressants 15. Ketamine 16. Ethyl chloride 17. Methadone 18. GHB 19. Anabolic steroids | 168 (51) | 23.1 | 5.6 | 18.4185 | 4.6639 | 3.6 | 2.6 | NR | NR | 1. Mean number of school years completed: 12.2 ±1 2. Trade or technical qualification: 85 (26)  3. University degree or college course: 75 (23) |  | 1. Employed full-time: 115 (35)  2. Students: 111 (34)  3. Unemployed: 52 (16) 4. Employed part-time or casually, or engaged in home duties: 49 (15) |  | 1. Indigenous Australian descent: 7 (2) | "The study concluded that ecstasy use is associated with significant hazards, particularly among young, female, polydrug users and those who binge on ecstasy. These individuals reported various problems, including physical, psychological, financial, relationship, and occupational issues attributed to their ecstasy use. The findings challenge the perception that ecstasy is a relatively benign drug, highlighting the need for credible information dissemination and tailored treatment options for affected users. A substantial portion of the sample expressed a desire to reduce their ecstasy use and sought formal treatment, indicating a clear demand for support services" |
| Voce 2019 | Cross-Sectional | Australia | Meth | 154 | 1. Meth: 154 (100) | 1. Tobacco  2. Cannabis 3. Alcohol 4. Benzodiazepines  5. Heroin | 48 (31) | 39.4 | 10.1 | 19.3 | 7.1 | 20.1 | 9.5 | NR | NR |  | 1. Single: 98 (64) | 1. Unemployed: 103 (67) | 1. Injection: 113 (74) 2. Smoking: 30 (20) | NR | "A negative symptom factor exists in methamphetamine users not meeting schizophrenia criteria, possibly linked to polysubstance use. Polysubstance use may obscure diagnostic differences between methamphetamine-associated psychosis and schizophrenia. The total sample size was corrected from 154 to 153 participants. The percentage of participants in the 'low-symptoms class' was corrected from 38% to 25%. The authors acknowledged errors in the original publication and apologized for any inconvenience." |
| Vu 2017 | Cross-Sectional | Vietnam | Polysub. | 622 | Amphetamine-Type-Stimulants (ATS): 189 (30.4) | 1. Alcohol 2. ketamine 3. Cannabis 4. Heroine | 0 (0) | 24.5554 | 5.4242 | NR | NR | NR | NR | NR | NR | 1. Up to secondary school: 108 (17.4) 2. High school and some vocational training: 177 (28.5) 3. University undergraduate degree or higher: 336 (54.1) |  | 1. Student: 146 (23.5) 2. Office-based job: 156 (25.1) 3. Service job: 92 (14.8) 4. Self-employed/Casual employment: 188 (30.2) 5. Unemployed: 40 (6.4) |  | NR | "The study concludes that there is a significant association between depression and both amphetamine-type stimulant (ATS) use and enacted homosexuality-related stigma among men who have sex with men (MSM) in Vietnam. It highlights that higher levels of enacted stigma correlate with increased depression rates, supporting the Minority Stress Theory. The findings suggest that interventions addressing mental health, substance use, and stigma are crucial for effective HIV prevention strategies tailored for MSM in Vietnam. Future research should utilize longitudinal designs to better understand the causal relationships among these factors" |
| Watanabe 2009 | Cross-Sectional | Japan | Inmates | 52 | 1. Meth: 50 (96) | 1. Methamphetamine  2. Alcohol  3. Cannabis  4. Methylenedioxymethamphetamine (MDMA)  5. Inhalant abuse | 0 (0) | 38 | 1.5 | NR | NR | 4.8 | 0.63 | NR | NR | 1. Years of education: 9.8 ±1.7 | 1. Never married: 9 (17.3) | 1. Full-time employment past 3 years: 40 (76.9) | 1. Injection: 41 (80.8) | NR | "Japanese methamphetamine abusers in correctional settings exhibit distinct characteristics compared to those in medical settings. Specific problems, such as mental health issues, need assessment for these abusers. The study highlights the necessity for tailored support facilities for correctional inmates. Findings indicate that the environmental backgrounds of these abusers differ significantly from those in hospitals." |
|  |  |  | Patients | 55 | 1. Meth: 52 (94.5)  2. MDMA: 6 (10.9) Abuse Period (y): 2.3 ± 0.56 |  | 0 (0) | 35.9 | 1.2 | NR | NR | 8.2 | 0.91 | NR | NR | 1. Years of education: 11.6 ±2.3 | 1. Never married: 36 (65.5) | 1. Full-time employment past 3 years: 24 (43.6) | 1. Injection: 23 (41.8) | NR |  |
| Watt 2015 | Cross-Sectional | South Africa | Meth | 360 | 1. Meth: 360 (100) | 1. Alcohol | 159 (44) | 28.973 | 7.3045 | NR | NR | 7.0617 | 3.6439 | NR | NR | 1. Completed secondary school: 42 (11.6) | 1. Currently married: 50 (13.8) | 1. Employed (part or full time): 67 (18.6) |  | 1. Colored v. black African: 263 (73) | "This study clearly demonstrates that mental health services should be made more readily available to methamphetamine users. The high prevalence of suicide attempts and psychological distress in this sample highlights the need for evidence-based substance abuse prevention interventions that also promote mental health through developing adaptive coping strategies. Additionally, to improve drug treatment outcomes, programmes should screen methamphetamine users for suicide risk and psychological distress, deliver services that treat their substance use and underlying mental health conditions in an integrated manner, and teach adaptive coping strategies to prevent relapse to substance use." |
| Wong 2013 | Cross-Sectional | USA | Polysub. | 73183 | 1. Methamphetamine: 4,611 (6.3) 2. Ecstasy: 5,928 (8.1) | 1. Heroin 2. Methamphetamine 3. Steroids 4. Cocaine 5. Inhalants 6. Hallucinogen 7. Ecstasy 8. Alcohol 9. Tobacco 10. Marijuana | 36079 (49.3) | NR | NR | NR | NR | NR | NR | NR | NR | 1. 9th Grade: 21,223 (29.00) 2. 10th Grade: 19,101 (26.10) 3. 11th Grade: 17,125 (23.40) 4. 12th Grade: 15,734 (21.50) |  |  |  | 1. White: 45227 (61.80) 2. Black: 10392 (14.20) 3. Hispanic: 7904 (10.80) 4. Multiracial: 5855 (8.00) 5. Asian: 2415 (3.30) 6. American Indian: 659 (0.90) 7. Pacific Islander: 585 (0.80) | "Substance abuse significantly increases the risk of suicidal thoughts and behaviors among adolescents. The strength of this relationship escalates with specific illicit drugs and higher substance use. Routine screening for substance abuse is crucial in assessing adolescent suicide risk" |
| Yen 2015 | Cross-Sectional | Taiwan | Had suicidal ideation | 32 | 1. Meth: 32 (100) 2. Amphetamine: 14 (43.8) | 1. Alcohol  2. Nicotine  3. Betel nut 4. Amphetamine | 19 (59.4) | 17.2 | 0.8 | 15.2 | 1.7 | NR | NR | NR | NR | 1. Education (y): 7.7 ± 1.5 |  | NR | NR | NR | "The study found that 16% of adolescent MAMP users experienced suicidal ideation in the past year. Factors such as family drug use and emotional instability correlate with suicidal ideation. Adolescent MAMP users with depressive or adjustment disorders should be closely monitored for suicidal ideation. The study emphasizes the importance of family dynamics over mere family structure in understanding suicidal behavior. Further research is needed to assess suicidal behaviors in adolescents who have never used MAMP." |
|  |  |  | Did not have suicidal ideation | 168 | 1. Meth: 168 (100) 2. Amphetamine: 51 (30.4) |  | 56 (33.3) | 17 | 0.8 | 15.7 | 1.4 | NR | NR | NR | NR | 1. Education (y): 8.4 ± 1.2 |  | NR | NR | NR |  |
| Yockey 2020 | Cross-Sectional | USA | Did Not Use Meth | 88,534 | 1. Ecstasy Use: 2267 (2.56) | 1. Alcohol 2. Marijuana 3. LSD 4. Ecstasy | 46774 (52.8) | 24.8 | 4.4 | NR | NR | NR | NR | NR | NR | NR | NR | NR | NR | 1. White: 49,038 (55.42) 2. Hispanic: 17,601 (19.89) 3. Black/African American: 12,015 (13.58) 4. Asian: 4,480 (5.06) 5. Multiracial: 3,522 (3.98) 6. Native American/Alaskan Native: 1,352 (1.53) 7. Native HI/Pacific Islander: 469 (0.53) | "Methamphetamine use remains a national health crisis that requires urgent attention and intervention. The study analyzed past-year methamphetamine use among young adults in the US from 2015 to 2018. Findings indicate low prevalence but significant comorbid drug use and behavioral health issues. Results can inform prevention efforts and harm reduction programs targeting at-risk populations." |
|  |  |  | Used Meth | 912 | 1. Meth: 912 (100) 2. Ecstasy Use: 150 (16.54) |  | 377 (41.3) | 25.33 | 4.35 | NR | NR | NR | NR | NR | NR | NR | NR | NR | NR | 1. White: 631 (66.63) 2. Hispanic: 144 (15.21) 3. Multiracial: 53 (5.60) 4. Black/African American: 43 (4.54) 5. Native American/Alaskan Native: 50 (5.28) 6. Asian: 17 (1.80) 7. Native HI/Pacific Islander: 9 (0.95) |  |

**Abbreviations:** Meth: Methamphetamine; AMPH: Amphetamine; MDMA: 3,4-Methylenedioxymethamphetamine (Ecstasy); Polysub.: Polysubstance use (multiple drugs); MIP: Meth-Induced Psychosis; GD: Gambling Disorder
