# Supplementary material for: Suicide and psychiatric disorders associated with amphetamine type stimulant use: a systematic review and meta-analysis
Source: Front Psychiatry. 2026 Mar 13;17:1654091. doi: 10.3389/fpsyt.2026.1654091 (PMC13022986; doi:10.3389/fpsyt.2026.1654091)
Supplement: SUPPLEMENTARY TABLE 4 — Risk of bias for cross-sectional and cohort studies. [file Table4.pdf]

Supplementary Table 4: Risk of bias for cross-sectional and cohort studies.

Study ID	NIH Quality Assessment Tool for Observational Cohort and Cross-Sectional Studies															Quality rating: good (11-14 points) or fair (7.5-10.5 points) or poor (0-7 points)
	1. Was the research question or objective in this paper clearly stated?	2. Was the study population clearly specified and defined?	3. Was the participation rate of eligible persons at least 50%?	4. Were all the subjects selected or recruited from the same or similar populations (including the same time period)? Were inclusion and exclusion criteria for being in the study prespecified and applied uniformly to all participants?	5. Was a sample size justification, power description, or variance and effect estimates provided?	6. For the analyses in this paper, were the exposure(s) of interest measured prior to the outcome(s) being measured?	7. Was the time frame sufficient so that one could reasonably expect to see an association between exposure and outcome if it existed?	8. For exposures that can vary in amount or level, did the study examine different levels of the exposure as related to the outcome (eg, categories of exposure, or exposure measured as continuous variable)?	9. Were the exposure measures (independent variables) clearly defined, valid, reliable, and implemented consistently across all study participants?	10. Was the exposure(s) assessed more than once over time?	11. Were the outcome measures (dependent variables) clearly defined, valid, reliable, and implemented consistently across all study participants?	12. Were the outcome assessors blinded to the exposure status of participants?	13. Was loss to follow-up after baseline 20% or less?	14. Were key potential confounding variables measured and adjusted statistically for their impact on the relationship between exposure(s) and outcome(s)?	total scores	
	Yes/ No / Not reported (NR) or cannot determine (CD) or not applicable (NA)	Yes/ No / Not reported (NR) or cannot determine (CD) or not applicable (NA)	Yes/ No / Not reported (NR) or cannot determine (CD) or not applicable (NA)	Yes/ No / Not reported (NR) or cannot determine (CD) or not applicable (NA)	Yes/ No / Not reported (NR) or cannot determine (CD) or not applicable (NA)	Yes/ No / Not reported (NR) or cannot determine (CD) or not applicable (NA)	Yes/ No / Not reported (NR) or cannot determine (CD) or not applicable (NA)	Yes/ No / Not reported (NR) or cannot determine (CD) or not applicable (NA)	Yes/ No / Not reported (NR) or cannot determine (CD) or not applicable (NA)	Yes/ No / Not reported (NR) or cannot determine (CD) or not applicable (NA)	Yes/ No / Not reported (NR) or cannot determine (CD) or not applicable (NA)	Yes/ No / Not reported (NR) or cannot determine (CD) or not applicable (NA)	Yes/ No / Not reported (NR) or cannot determine (CD) or not applicable (NA)	Yes/ No / Not reported (NR) or cannot determine (CD) or not applicable (NA)		
**Akiyama 2006**	Yes	Yes	No	Yes	No	Yes	Yes	Yes	Yes	NR	Yes	NR	NR	No	9.5	Fair
**Akiyama 2011**	Yes	Yes	No	Yes	Yes	Yes	Yes	Yes	Yes	No	Yes	CD	Yes	No	11.5	Good
**Argento2017**	Yes	Yes	Yes	Yes	No	Yes	No	Yes	Yes	No	Yes	CD	Yes	No	11	Good
**Attafi 2021**	Yes	Yes	Yes	Yes	Yes	Yes	Yes	No	Yes	Yes	Yes	CD	Yes	No	12	Good
**Auten 2012**	Yes	No	No	Yes	No	Yes	No	Yes	Yes	No	Yes	CD	Yes	No	10	Fair
**Chen 2021**	Yes	Yes	No	Yes	No	Yes	Yes	No	Yes	No	Yes	CD	No	No	10	Fair
**Coffin 2024**	Yes	Yes	No	Yes	No	Yes	Yes	No	Yes	Yes	Yes	CD	CD	No	10	Fair
**Darke 2017**	Yes	Yes	No	Yes	No	Yes	Yes	Yes	Yes	No	Yes	CD	CD	No	10	Fair
**Darke 2018**	Yes	Yes	No	Yes	No	Yes	Yes	Yes	Yes	No	Yes	CD	CD	No	10	Fair
**Dixson 2017**	Yes	Yes	Yes	Yes	No	NA	NA	Yes	Yes	NA	Yes	CD	NA	Yes	8.5	Fair
**Domier 2000**	Yes	Yes	No	Yes	No	Yes	Yes	Yes	Yes	No	Yes	CD	NA	No	10	Fair
**Ericsson 2013**	Yes	Yes	No	Yes	No	Yes	Yes	Yes	Yes	No	Yes	CD	NR	Yes	10.5	Fair
**Fang 2023**	Yes	Yes	No	Yes	No	Yes	Yes	Yes	Yes	No	Yes	CD	NR	Yes	10.5	Fair
**Fatovich 2010**	Yes	Yes	No	Yes	No	NA	NA	Yes	Yes	NA	Yes	CD	NA	No	7.5	Fair
**Fletcher 2019**	Yes	Yes	No	Yes	No	NA	NA	Yes	Yes	NA	Yes	CD	NA	No	7.5	Fair
**Gardner 1972**	Yes	Yes	No	Yes	No	NA	NA	Yes	Yes	NA	Yes	NR	NA	No	7.5	Fair
**Gonzales 2011**	Yes	Yes	No	Yes	No	NA	NA	Yes	Yes	NA	Yes	NR	NA	Yes	8	Fair
**Hajebi 2016**	Yes	Yes	No	Yes	No	Yes	Yes	NA	Yes	No	Yes	NR	Yes	No	10	Fair
**Herbeck 2015**	Yes	Yes	No	Yes	No	Yes	Yes	No	Yes	No	Yes	CD	Yes	No	10.5	Fair
**Karch 1985**	Yes	Yes	No	Yes	No	Yes	Yes	Yes	Yes	No	Yes	CD	NA	No	10	Fair
**Kaye 2008**	Yes	Yes	No	Yes	No	Yes	Yes	Yes	Yes	No	Yes	CD	NA	No	10	Fair
**Kaye 2009**	Yes	Yes	No	Yes	No	Yes	Yes	Yes	Yes	No	Yes	CD	NA	Yes	10.5	Fair
**Kittirattanapaiboon 2010**	Yes	Yes	No	Yes	No	Yes	Yes	Yes	Yes	No	Yes	CD	No	Yes	11	Good
**Kuo 2010**	Yes	Yes	NA	Yes	No	Yes	Yes	No	Yes	No	Yes	Yes	NA	Yes	10.5	Fair
**Lee 2021**	Yes	Yes	NA	Yes	No	NA	NA	Yes	Yes	NA	Yes	CD	NA	Yes	7.5	Fair

Supplementary Table 4: Risk of bias for cross-sectional and cohort studies.

**Marshall 2011**	Yes	Yes	No	Yes	No	Yes	Yes	Yes	Yes	Yes	Yes	CD	NR	Yes	11	Good
**Marshall 2012**	Yes	Yes	No	Yes	No	Yes	Yes	Yes	Yes	Yes	Yes	CD	NR	Yes	11	Good
**Mcketin 2020**	Yes	Yes	No	Yes	No	Yes	Yes	Yes	Yes	NR	Yes	CD	NR	Yes	10	Fair
**Paknahad 2020**	Yes	Yes	No	Yes	No	NA	Yes	No	Yes	No	Yes	NR	NA	No	8.5	Fair
**Paydar 2015**	Yes	Yes	No	Yes	No	NA	Yes	No	Yes	No	Yes	NR	NA	No	8.5	Fair
**Rawson 2005**	Yes	Yes	No	Yes	No	Yes	Yes	No	Yes	No	Yes	NR	NR	Yes	10	Fair
**Trombello 2023**	Yes	Yes	No	Yes	No	Yes	Yes	No	Yes	Yes	Yes	NR	NR	Yes	10.5	Fair
**Zarrabi 2016**	Yes	Yes	No	Yes	No	NA	NA	No	Yes	NA	Yes	NR	NA	No	7	Poor
**Ali 2024**	Yes	Yes	Yes	Yes	Yes	NA	NA	No	Yes	NA	Yes	No	NA	No	8.5	Fair
**Al-Imam 2023**	Yes	Yes	No	Yes	Yes	NA	NA	Yes	Yes	NA	Yes	NR	NA	Yes	8.5	Fair
**Artenie 2014**	Yes	Yes	No	Yes	No	NA	NA	Yes	Yes	NA	Yes	NR	NA	Yes	8	Fair
**Baberg 1995**	Yes	Yes	No	Yes	No	NA	NA	No	Yes	NA	Yes	NR	NA	Yes	7.5	Fair
**Brecht 2004**	Yes	Yes	Yes	Yes	No	NA	NA	Yes	Yes	NA	Yes	NR	NA	Yes	8.5	Fair
**Chen 2007**	Yes	Yes	No	Yes	No	NA	NA	Yes	Yes	NA	Yes	NR	NA	Yes	8	Fair
**Christian 2007**	Yes	Yes	Yes	Yes	No	NA	NA	Yes	Yes	NA	Yes	NR	NA	Yes	8.5	Fair
**Cloutier 2013**	Yes	Yes	No	Yes	No	NA	NA	No	Yes	NA	Yes	NR	NA	Yes	7.5	Fair
**Demir 2021**	Yes	Yes	No	Yes	No	NA	NA	Yes	Yes	NA	Yes	NR	NA	Yes	8	Fair
**Derlet 1988**	Yes	Yes	No	Yes	No	NA	NA	No	Yes	NA	Yes	NR	NA	No	7	Poor
**Fass 2009**	Yes	Yes	No	Yes	No	NA	NA	Yes	Yes	NA	Yes	NR	NA	No	7.5	Fair
**Frankeberger 2024**	Yes	Yes	Yes	Yes	No	NA	NA	Yes	Yes	NA	Yes	NR	NA	No	8	Fair
**Hadinezhad 2018**	Yes	Yes	Yes	Yes	No	NA	NA	No	Yes	NA	Yes	NR	NA	No	7.5	Fair
**Hypse 2018**	Yes	Yes	No	Yes	No	NA	NA	No	Yes	NA	Yes	NR	NA	Yes	7.5	Fair
**Kalayasiri 2009**	Yes	Yes	No	Yes	No	NA	NA	Yes	Yes	NA	Yes	NR	NA	Yes	8	Fair
**Kalechstein 2000**	Yes	Yes	Yes	Yes	No	NA	NA	No	Yes	NA	Yes	NR	NA	Yes	8	Fair
**Karabulut 2023**	Yes	Yes	No	Yes	Yes	NA	NA	Yes	Yes	NA	Yes	NR	NA	Yes	8.5	Fair
**Li 2023**	Yes	Yes	Yes	Yes	Yes	NA	NA	Yes	Yes	NA	Yes	NR	NA	Yes	9	Fair
**Lin 2004**	Yes	Yes	No	Yes	No	NA	NA	Yes	Yes	NA	Yes	NR	NA	Yes	8	Fair
**Massah 2019**	Yes	Yes	Yes	Yes	No	NA	NA	No	Yes	NA	Yes	NR	NA	No	7.5	Fair
**McKetin 2011**	Yes	Yes	Yes	Yes	No	NA	NA	Yes	Yes	NA	Yes	NR	NA	No	8	Fair
**Miller 2016**	Yes	Yes	No	Yes	No	NA	NA	Yes	Yes	NA	Yes	NR	NA	No	7.5	Fair
**Nazari 2023**	Yes	Yes	No	Yes	No	NA	NA	Yes	Yes	NA	Yes	NR	NA	No	7.5	Fair
**Njuguna 2021**	Yes	No	No	Yes	No	NA	NA	No	Yes	NA	Yes	NR	NA	No	6.5	Poor
**Park 2022**	Yes	Yes	No	Yes	No	NA	NA	Yes	Yes	NA	Yes	NR	NA	No	7.5	Fair
**Peng 2025**	Yes	Yes	Yes	Yes	Yes	NA	NA	No	Yes	NA	Yes	No	NA	Yes	9	Fair
**Perello 2022**	Yes	Yes	Yes	Yes	No	NA	NA	Yes	Yes	NA	Yes	NR	NA	No	8	Fair
**Richards 1999**	Yes	Yes	Yes	Yes	No	NA	NA	No	Yes	NA	Yes	NR	NA	No	7.5	Fair
**Richards 2017**	Yes	Yes	No	Yes	No	NA	NA	No	Yes	NA	Yes	NR	NA	Yes	7.5	Fair
**Roxburgh 2020**	Yes	No	No	Yes	No	NA	NA	No	Yes	NA	Yes	NR	NA	No	6.5	Poor
**Soboka 2024**	Yes	Yes	No	Yes	Yes	NA	NA	Yes	Yes	NA	Yes	NR	NA	Yes	8.5	Fair
**Stronach 2024**	Yes	No	No	No	Yes	NA	NA	No	Yes	NA	Yes	NR	NA	No	6.5	Poor
**Toles 2006**	Yes	No	No	Yes	No	NA	NR	No	Yes	NR	Yes	NR	NA	Yes	7	Poor
**Topp 1998**	Yes	Yes	No	Yes	No	NA	NA	No	Yes	NA	Yes	No	NA	No	7.5	Fair
**Voce 2019**	Yes	Yes	No	Yes	Yes	NA	NA	No	Yes	NA	Yes	NR	NA	Yes	8	Fair
**Vu 2017**	Yes	Yes	No	Yes	No	NA	NA	No	Yes	NA	Yes	No	NA	No	7.5	Fair
**Wang 2025**	Yes	No	No	Yes	Yes	NA	NA	No	Yes	NA	Yes	NR	NA	No	7	Poor
**Watanabe 2009**	Yes	Yes	Yes	Yes	Yes	NA	NA	No	Yes	NA	Yes	NR	NA	No	8	Fair
**Watt 2015**	Yes	Yes	Yes	Yes	No	NA	NA	No	Yes	NA	Yes	NR	NA	Yes	8	Fair
**Wei 2023**	Yes	No	No	Yes	No	NA	NA	No	Yes	NA	Yes	NR	NA	Yes	7	Poor
**Wong 2013**	Yes	Yes	No	Yes	No	NA	NA	No	Yes	NA	Yes	NR	NA	No	7	Poor
**Yen 2005**	Yes	Yes	No	Yes	No	NA	NA	No	Yes	NA	Yes	NR	NA	Yes	7.5	Fair
**Yockey 2020**	Yes	Yes	No	Yes	Yes	NA	NA	No	Yes	NA	Yes	NR	NA	Yes	8	Fair
